# The effect of endophytic bacteria on the growth, medicinal quality, and rhizosphere soil environment of *Isatis indigotica* Fort

**DOI:** 10.3389/fpls.2026.1821717

**Published:** 2026-04-22

**Authors:** Yuqin Hu, Liqiong Sun, Xiaofan Li, Min Yang, Xiaoqing Tang, Kangcai Wang

**Affiliations:** 1 College of Horticulture, Nanjing Agricultural University, Nanjing, China; 2 Jiangsu Qihuang Tiancheng Health Industry Group Co., Ltd., Changshu, China

**Keywords:** endophytic bacteria, growth, *Isatis indigotica*, medicinal quality, rhizosphere soil environment, soil microbial diversity

## Abstract

Plant growth-promoting endophytes (PGPE) can form a mutually beneficial symbiotic relationship with host plants, analyzing the ability of endophytic bacteria of *Isatis indigotica* to promote growth and improve the rhizosphere environment and exploring the influence of dominant endophytic bacteria on the structure of rhizosphere microbial communities. In this study, we evaluated the ability of the three endophytic bacteria strains by a field experiment. The single endophytic bacterial strain and combination of every two bacterial strains were used for irrigating the rhizosphere of *I. indigotica* four times, and related indicators and rhizosphere soil of *I. indigotica* were measured. We screened out the dominant treatment groups based on the total active biomass of *I. indigotica* and analyzed microbial diversity of rhizosphere soil in dominant treatment groups. The results showed that endophyte treatments had significant effects on growth and physiology of *I. indigotica*, in which T11–28 and BC00 had the most significant effect on the dry weight of the aboveground part and underground part, respectively. The endophyte treatments had different effects on the content of active ingredients, rhizosphere soil chemical properties, and enzyme activities of *I. indigotica*, with BC00 promoting indigo and indirubin in leaves most significantly and BV11 promoting epigoitrin in roots most effectively. Total active biomass was calculated as the product of active ingredient content and biomass per plant. Based on this parameter, BC00 was the dominant treatment group, and the analysis of the diversity of its rhizosphere soil flora revealed that BC00 was able to enrich *Methylobacillus*, *Alternaria*, and other plant-growth-friendly flora. In the comprehensive analysis, the treatments of three endophytic bacterial strains of *I. indigotica* had significant promotion effects on its growth physiology and active ingredients and had obvious improvement effects on the rhizosphere environment, among which BC00 had the best comprehensive effect, which was associated with alterations in the rhizosphere soil microbial community structure.

## 1 Introduction

Medicinal plants are natural resources with important medicinal values. In order to meet people’s demand for high yields, the irrational use of fertilizers and medicines has become a common phenomenon, resulting in poor quality of herbal medicines and threatening the soil environment due to continuous cropping obstacles and soil compaction. In order to minimize the adverse effects of intensive production and long-term continuous cropping cultivation mode, innovative methods of regulation through microorganisms have gained greater attention in recent years, and plants and microorganisms form a symbiotic relationship in which microorganisms can influence plant growth and health and further improve soil quality and nutrient cycling effectively (Hassan, 2017). With the promotion of policies such as the “double reduction” of chemical fertilizers and pesticides and the ecology of traditional Chinese medicine agriculture, the application of microbial fertilizers has gradually become a hotspot (Lai et al., 2024). The application of plant endophytes as biocontrol agents and plant growth promoters has received more and more attention, which can improve the fertilization efficiency of microbial fertilizers and is also an important microbial fertilizer strain resource, and has not been fully developed (Falade et al., 2021; De Vries et al., 2020; Kong et al., 2019). Plant growth-promoting endophytes (PGPE) can promote plant growth by producing growth-promoting hormones, providing nitrogen, and dissolving phosphate (Lin and Xu, 2013; Matsuoka et al., 2013; Shi et al., 2011). Research on the growth-promoting effect and rhizosphere environment improvement ability of endophytes can provide a reference for the development of special microbial fertilizers for medicinal plants, which can further promote the green and healthy development of the Chinese herbal medicine industry.

*Isatis indigotica* Fort. is a biennial herb of the genus *Isatis* of the Cruciferae family, which is cold in nature, bitter in taste, and has the efficacy of clearing heat and detoxifying, cooling blood to resolve macules, and relieving sore throat and pain (National Pharmacopoeia Commission, 2025). *I. indigotica* is rich in alkaloids, organic acids, flavonoids, lignans, nucleosides, steroids, and amino acids, which have pharmacological activities such as antiviral, antibacterial, anti-endotoxin, anti-inflammatory, antitumor, and immunostimulatory (Yu et al., 2018; Chen et al., 2021; Zhou and Zhang, 2013). According to the Pharmacopoeia of the People’s Republic of China (2025 Edition) (National Pharmacopoeia Commission, 2025), indigo, indirubin, and epigoitrin are stipulated as the key quality markers for evaluating the medicinal materials of *I. indigotica* leaf and root, respectively. Therefore, obtaining high-yield and high-quality *Isatis* root and *Isatis* leaf herbs is the main goal of standardized production of *I. indigotica*, but the phenomenon of large amounts of fertilizers still exists in its cultivation and management, and the excessive pursuit of yield has led to a decline in the quality of herbs for medicinal purposes, and “lose weight and improve quality” is imperative. Plant endophytes are a class of special “microorganisms” with important value, which can parasitize different parts of living plants but will not obviously cause symptoms of external infestation of the host plant or a harmonious coexistence with the host (Chen et al., 2022), such as *Atractylodes macrocephala* inoculated with endophytic bacterium *Pseudomonas* ALEB7B, the accumulation of its sesquiterpenoids increased significantly (Zhou et al., 2018). In line with China’s agricultural green development policies promoting precision fertilization and scientific pesticide application, three strains of endophytic bacteria isolated from the roots of *I. indigotica* in the early stage of the experiment, which have good plant growth-promoting activities, were used in single endophytic bacterial strain and combination of every two bacterial strains treatments to irrigate the roots with bacterial suspensions to explore the effects of these strains on the growth of *I. indigotica* and its rhizosphere environment, and to analyze the effects of dominant endophytic bacteria on the structure of the rhizosphere microbial community.

## 2 Materials and methods

### 2.1 Materials

The endophytic bacteria *Bacillus* sp. (BC00), *Bacillus* sp. (BV11), and *Pseudomonas* sp. (PA28) were previously isolated from the roots of *I. indigotica* using the streak plate method. The test plant material was *I. indigotica* silique, which was from Shandong Province and was identified as the cruciferous plant *Isatis indigotica* Fort.

### 2.2 Experimental design

The endophytic bacteria for the test were activated and cultured, inoculated into LB liquid medium, incubated at 180 r·min^-1^ for 72 h in a constant-temperature shaking incubator at 28 °C, followed by centrifugation at 4000 r·min^-1^ for 10 min to collect the bacterial cells, which were washed three times with PBS (pH 7.4) and resuspended in sterile water to a single bacterial suspension at a concentration of 1.0×10^8^ CFU·mL^-1^; the composite bacterial solution was mixed with an equal volume of the single bacterial solution.

The experiment was carried out at the Changshu Shanghu Chinese medicinal herb base of Jiangsu Qihuang Tiancheng Health Industry Group Co., Ltd., and *I. indigotica* was sown on April 11, 2024. A total of seven treatments were established, in which the control group (CK) was applied with sterile water, and the experimental groups were single-strain treatments BC00, BV11, and PA28 and combinations of two-strains treatments BC00+BV11 (T00-11), BC00+PA28 (T00-28), and BV11+PA28 (T11-28), respectively. Three plots for each treatment, a total of 21 plots, with a plot size of 1.2 m×5 m, were planted in a randomized block design, and the plots were planted in a north-south row orientation. From June to September, each plant was irrigated with 50 mL of bacterial suspension per root once per month (four times in total), and the control group was treated with an equal amount of sterile water. The 30-day irrigation interval was determined based on previous studies from our research group.

### 2.3 Photosynthetic parameter measurements

Under sunny and clear weather conditions, the middle and upper parts of the third round of leaves of *I. indigotica*, which were fully expanded and unbroken from the inside out, were selected as the measurement site from 9:00 to 11:00 on December 15 of the same year, and the net photosynthetic rate (*Pn*), the stomatal conductance (*Gs*), the intercellular CO_2_ concentration (*Ci*) and the transpiration rate (*Tr*) of the leaves were measured using an LI-6800 portable photosynthesis system with a light intensity of 1200 μmol·m^−2^·s^−1^, and each treatment was repeated 15 times. Water use efficiency (WUE) was calculated according to Equation 1:

(1) Water use efficiency=Pn/Tr

### 2.4 Growth parameter measurements

*I. indigotica* was harvested on December 15 of the same year, and randomly selected fresh plant samples were preserved at -80 °C. The plant height (length from the base to the tip of the longest leaf), the number of leaves, the length and diameter of the taproot, and the number of lateral roots were measured, and the fresh weights of the shoot and root parts were weighed. The samples were placed in a 105 °C oven for 20 min for enzyme inactivation, then dried at 60 °C to constant weight. The dry weights of shoot and root parts were weighed, and the root-shoot ratio was calculated according to Equation 2:

(2) Root−shoot ratio=dry weight of root/dry weight of shoot

After drying, the leaves and roots were separately ground and sieved through a 60-mesh sieve for determination of active ingredient contents.

### 2.5 Photosynthetic pigment measurements

Three plants were randomly selected from the apical 1/3 of the third whorl of functional leaves of *I. indigotica* from inside to outside, and the chlorophyll a, chlorophyll b, and carotenoid contents of leaves were determined by the ethanol extraction method (Wellburn, 1994).

### 2.6 Determination of key enzyme activities in nutrient metabolism

The leaves were selected in the same way as in section 2.5. Sucrose phosphate synthase (SPS) and nitrate reductase (NR) activities in *I. indigotica* leaves were determined using assay kits from Beijing Solarbio Science & Technology Co., Ltd. Acid phosphatase (ACP) activity was measured using kits from Shanghai Yuanye Bio-Technology Co., Ltd.

### 2.7 Determination of soluble substance content

The soluble protein content of leaves and roots was determined by using the Coomassie Brilliant Blue G-250 method (Morris, 1948), and the soluble sugar and soluble starch content of leaves and roots were determined by using the anthrone colorimetric method (Morris, 1948; Maness, 2010).

### 2.8 Determination of endogenous hormone content

Indole-3-acetic acid (IAA), gibberellic acid 3 (GA_3_), abscisic acid (ABA), and zeatin riboside (ZR) were determined by enzyme-linked immunosorbent assay in leaves and roots, and the ELISA kits were purchased from Shanghai Yuanju Biotechnology Center.

### 2.9 Determination of active ingredient content

The total flavonoid content was determined by colorimetric method using rutin as a control (Guan et al., 2018). The peak areas (*y*) and concentrations (*x*, μg·mL^-1^) of the standard curves were plotted by high-performance liquid chromatography (HPLC) with slight modifications with reference to the Pharmacopoeia of the People’s Republic of China (General Rule 0512) for the determination of indigo and indirubin in leaves and epigoitrin in roots (National Pharmacopoeia Commission, 2025). The regression equations for indigo, indirubin, and epigoitrin were *y_1_* = 5.33×10^4^*x_1_* + 3.66×10^4^ (*R²* = 0.9997, *n* = 3), with a linear range of 10-60 μg·mL^-1^, *y_2_* = 8.90×10^4^*x_2_* + 1.15×10^4^ (*R²* = 0.9994, *n* = 3), with a linear range of 2-40 μg·mL^-1^, and *y_3_* = 1.15×10^5^*x_3_*-3.30×10^5^ (*R²* = 0.9930, *n* = 3), with a linear range of 10-50 μg·mL^-1^.

### 2.10 Determination of rhizosphere soil chemical properties

The rhizosphere soil was collected after harvesting *I. indigotica*, and part of the fresh samples were kept at -80 °C, while the rest were air-dried indoors, ground through a 100-mesh sieve, and set aside. Soil pH was determined by the potentiometric method (Vogel et al., 2023), soil alkali-hydrolyzable nitrogen by the alkali-hydrolyzable diffusion method (Bao, 2000), soil available phosphorus by the sodium bicarbonate extraction-molybdenum-antimony anti-colorimetric method (Olsen, 1954), and soil available potassium by inductively coupled plasma optical emission spectrometry (Jiao et al., 2015).

### 2.11 Determination of rhizosphere soil enzyme activities

Soil catalase activity (S-CAT), soil urease activity (S-UE), soil nitrate reductase activity (S-NR), and soil sucrase activity (S-SC) were detected by using the kits of Beijing Solarbio Science & Technology Co., Ltd.

### 2.12 Correlation analysis and comprehensive analysis

Soil physicochemical properties, enzyme activities, and physiological indices of *I. indigotica* and its growth parameters and active components were selected for correlation analysis using the Pearson correlation coefficient method. The total active biomass per plant of *I. indigotica* was calculated according to Equation 3. To objectively integrate both biomass yield and active ingredient accumulation, total active biomass per plant was calculated according to Equation 3. Based on this metric, the superior treatment group was selected for subsequent analysis of rhizosphere soil microbial diversity.

(3) Total active biomass per plant=Active ingredient content per plant × Biomass per plant

### 2.13 Rhizosphere soil microbial diversity analysis

Illumina NovaSeq high-throughput sequencing was used to examine the community structure of bacteria and fungi in the soil of the dominant treatment group.

### 2.14 Correlation analysis between microorganisms and soil-plant indicators

Correlation analysis between selected key differential microorganisms and soil-plant indicators was performed following the method described in section 2.12.

### 2.15 Statistical analysis

The data were statistically analyzed using Microsoft Excel 2019, SPSS 27.0, R language, and a significance test of differences using Duncan’s new multiple range test with a significance level of 0.05, with *P*<0.05 representing a significant difference, and plotted using Origin 2025 and Adobe Illustrator 2024. In both tables and figures, quantitative data are presented as the mean ± standard deviation (SD). In figures, error bars represent the SD.

## 3 Results

### 3.1 Effects of endophytes on *I. indigotica* photosynthetic parameters

The *Pn*, *Gs*, and *Tr* were significantly higher than that of the CK (*P*<0.05), and the *Ci* concentration was significantly lower than that of the CK (*P*<0.05) in all treatment groups except for the T00–28 group, in which the WUE of the T11–28 treatment group was the highest and the *Pn*, *Gs*, and *Tr* of BV11 were the highest (**Table 1**).

**Table 1:** Effects of different treatments on photosynthetic parameters of *I. indigotica* (mean ± SD, *n*=15).

Treatment	*Pn*/μmol·m^-2^·s^-1^	*Gs*/mol·m^-2^·s^-1^	*Ci*/μmol·m^-2^·s^-1^	*Tr*/mmol·m^-2^·s^-1^	WUE/μmol·mmol^-1^
CK	4.831 ± 0.083f	0.025 ± 0.003e	337.740 ± 1.342b	1.139 ± 0.003e	4.239 ± 0.065b
BC00	6.043 ± 0.041c	0.072 ± 0.004b	317.593 ± 1.275e	1.823 ± 0.008b	3.315 ± 0.028d
BV11	7.569 ± 0.097a	0.130 ± 0.006a	292.193 ± 1.493g	2.484 ± 0.029a	3.047 ± 0.044e
PA28	5.516 ± 0.060d	0.040 ± 0.003c	322.733 ± 1.645d	1.453 ± 0.015c	3.796 ± 0.058c
T00-11	5.023 ± 0.009e	0.026 ± 0.003e	325.920 ± 3.168c	1.305 ± 0.018d	3.852 ± 0.052c
T00-28	3.089 ± 0.011g	0.018 ± 0.003f	353.517 ± 1.562a	1.107 ± 0.004f	2.791 ± 0.015f
T11-28	6.685 ± 0.049b	0.036 ± 0.007d	306.287 ± 2.730f	1.451 ± 0.079c	4.619 ± 0.247a

Different lowercase letters indicate significant difference among treatments (*P*<0.05), the same as below.

### 3.2 Effects of endophytes on photosynthetic pigments in *I. indigotica* leaves

**Figure 1:**
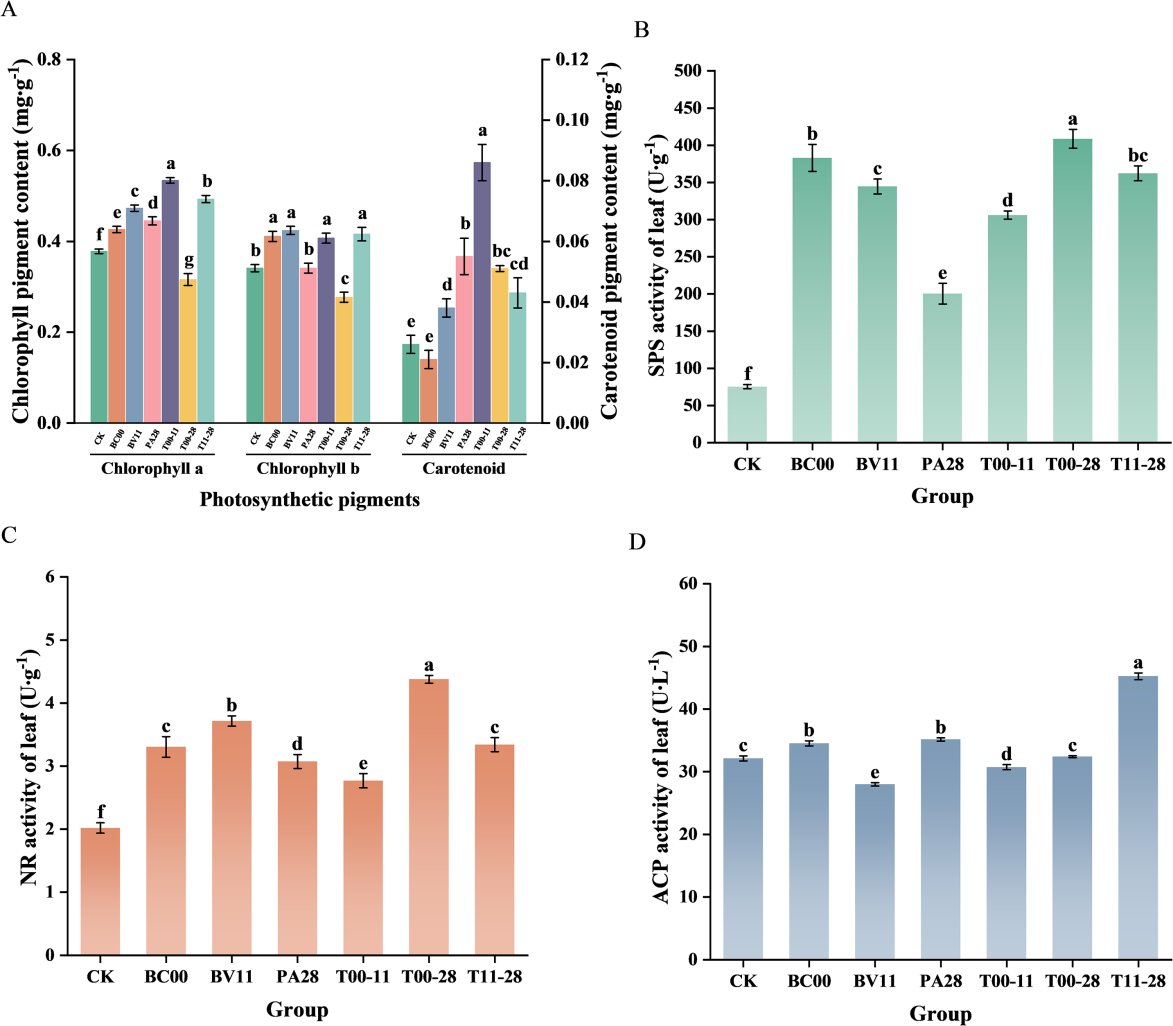
Photosynthetic pigment content and key enzyme activities in leaves of *I. indigotica* under different treatments (*n* = 3). **(A)** Photosynthetic pigment content, **(B)** Sucrose phosphate synthase (SPS), **(C)** Nitrate reductase (NR), **(D)** Acid phosphatase (ACP), Different colors represent different treatment groups, Different lowercase letters indicate significant difference among treatments (*P*<0.05), the same as below.

Compared with CK, the chlorophyll a and chlorophyll b contents were significantly higher (*P*<0.05) than those of CK except for the chlorophyll a of the T00–28 group and the chlorophyll b of PA28 and T00-28, in which T00–11 raised the chlorophyll a content by 41.27% and BV11 raised the chlorophyll b content by 24.34%, respectively. The carotenoid content of all treatment groups was significantly higher (*P*<0.05) than those of CK except BC00, in which T00–11 raised the content by 230.77% (**Figure 1A**).

### 3.3 Effects of endophytes on *I. indigotica* growth indicators

Plant height, number of blades, taproot diameter, length of taproot, and number of lateral roots were significantly higher (*P*<0.05) than those of CK in all treatment groups except for the T00–28 taproot length. Among them, T11–28 had the best effect on plant height promotion, which was 64.81% higher than that of CK. PA28 had the most significant effect on the number of blades and lateral roots, which were 4.53 and 2.25 times higher than those of CK, respectively, and BV11 had the most obvious effect on the promotion of taproot length, which was 24.27% higher than that of CK (**Table 2**).

**Table 2:** Differences in growth traits of *I. indigotica* under different treatments (mean ± SD, *n* = 10).

Treatment	Plant height/cm	Number of blades	Taproot length/cm	Taproot diameter/mm	Number of lateral roots
CK	9.69 ± 1.18d	16.50 ± 3.24e	11.99 ± 0.96de	1.18 ± 0.18c	5.50 ± 2.07c
BC00	14.24 ± 1.76c	41.50 ± 7.63d	13.23 ± 0.70bc	2.17 ± 0.27a	9.90 ± 2.69b
BV11	14.77 ± 0.76abc	45.80 ± 7.28cd	14.90 ± 1.59a	1.80 ± 0.52b	7.90 ± 1.66b
PA28	15.68 ± 1.31ab	74.70 ± 8.17a	12.98 ± 0.49bcd	2.37 ± 0.16a	12.40 ± 1.65a
T00-11	14.34 ± 1.22bc	48.70 ± 4.03c	12.36 ± 1.01cde	2.30 ± 0.07a	8.90 ± 1.66b
T00-28	14.49 ± 1.22bc	45.10 ± 5.22cd	11.44 ± 0.81e	2.11 ± 0.20a	9.40 ± 1.35b
T11-28	15.97 ± 0.72a	61.30 ± 3.16b	13.70 ± 0.75b	2.39 ± 0.17a	9.20 ± 1.03b

Different lowercase letters indicate statistically significant differences among treatments at the p<0.05 level.

The shoot fresh weight, root fresh weight, single plant fresh weight, shoot dry weight, root dry weight, and single plant dry weight of each treatment group were all significantly higher than those of the control (*P*<0.05). Compared with the CK, T11–28 had the most significant effect on shoot fresh weight and root dry weight, which were increased by 3.37-fold and 3.60-fold, and BC00 had the best effect on root fresh weight, single plant fresh weight, root dry weight, and single plant dry weight, which were increased by 4.25-fold, 3.23-fold, 6.12-fold, and 3.91-fold, respectively. Among the endophyte treatment groups, the root-shoot ratios of BC00 and BV11 were the highest and lowest, respectively (**Table 3**).

**Table 3:** Differences in biomass accumulation of single plant of *I. indigotica* under different treatments (mean ± SD, *n* = 10).

Treatment	Shoot fresh weight/g	Root fresh weight/g	Fresh weight/g	Shoot dry weight/g	Root dry weight/g	Dry weight/g	Root-shoot ratio
CK	36.50 ± 7.62e	21.69 ± 3.96f	58.19 ± 7.88e	7.30 ± 1.36d	5.48 ± 1.01f	12.78 ± 1.51f	0.78 ± 0.23c
BC00	95.60 ± 7.60bc	92.12 ± 5.34a	187.72 ± 7.25a	16.41 ± 1.46c	33.53 ± 1.94a	49.94 ± 1.71a	2.06 ± 0.28a
BV11	91.30 ± 10.62c	38.27 ± 3.10e	129.57 ± 13.20d	19.98 ± 2.29b	11.09 ± 0.90e	31.07 ± 3.01e	0.56 ± 0.04d
PA28	99.63 ± 4.63b	52.86 ± 4.97d	152.50 ± 7.88c	20.43 ± 0.95b	15.51 ± 1.46d	35.93 ± 1.99d	0.76 ± 0.07c
T00-11	100.99 ± 4.12b	73.11 ± 6.77b	174.10 ± 6.21b	18.98 ± 0.77b	20.02 ± 2.18b	39.01 ± 2.02c	1.06 ± 0.14b
T00-28	78.17 ± 3.34d	56.77 ± 3.19cd	134.94 ± 5.84d	19.07 ± 0.82b	15.56 ± 0.88d	34.63 ± 1.51d	0.82 ± 0.04c
T11-28	122.84 ± 4.21a	61.75 ± 6.93c	184.59 ± 10.91a	26.29 ± 0.9a	17.54 ± 1.97c	43.82 ± 2.81b	0.67 ± 0.06cd

Different lowercase letters indicate statistically significant differences among treatments at the p<0.05 level.

### 3.4 Effects of endophytes on nutrient metabolism enzymes in *I. indigotica* leaves

Compared with CK, the activities of SPS and NR in all treatment groups were significantly stronger than that of CK (*P*<0.05), in which T00–28 had the most significant promotion effect, with a 5.43-fold and 2.17-fold enhancement, respectively. Among the treatment groups, BC00, PA28, and T11–28 had a significant promotion effect on ACP activity (*P*<0.05), in which T11–28 having the best effect, with a 1.41-fold enhancement compared with that of CK (**Figures 1B–D**).

### 3.5 Effects of endophytes on *I. indigotica* soluble substance

**Figure 2:**
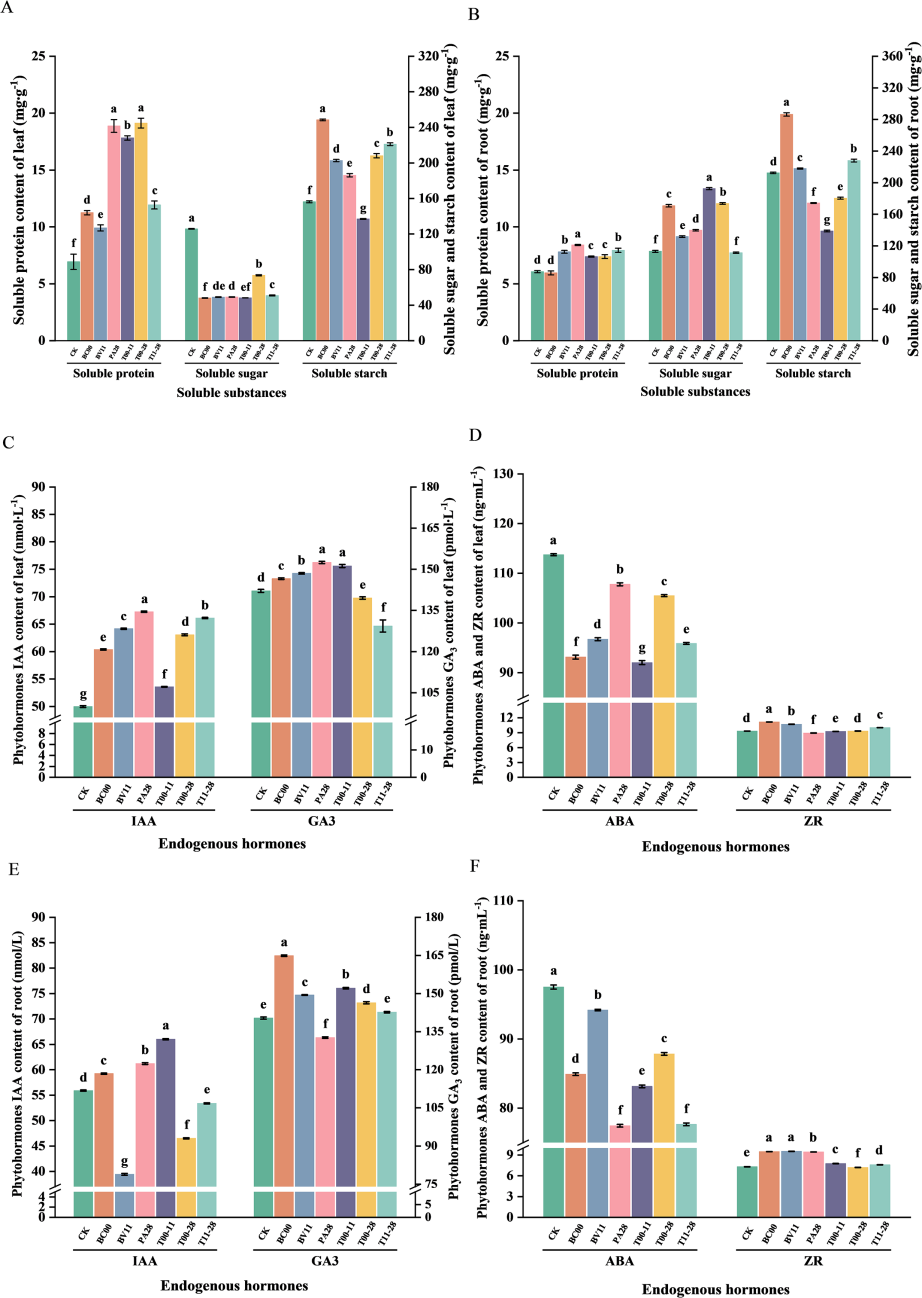
Soluble substance and endogenous hormone content of *I. indigotica* under different treatments (*n* = 3). **(A)** Soluble substance content in leaves, **(B)** Soluble substance content in roots, **(C)** IAA, GA_3_ content in leaves, **(D)** ABA, ZR content in leaves, **(E)** IAA, GA_3_ content in roots, **(F)** ABA, ZR content in roots.

Compared with CK, the soluble sugar contents of leaves in all treatment groups were significantly lower than that of the CK (*P*<0.05), and the soluble protein contents in leaves were significantly higher than that of CK (*P*<0.05), in which PA28 and T00–28 raised contents of soluble protein by 2.72-fold and 2.76-fold, respectively. Except for the T00–11 group, the content of soluble starch in leaves had a significant effect (*P*<0.05), among which BC00 promoted the effect most significantly, enhancing it 1.97-fold compared with CK. The root soluble protein content of BC00 was not significantly different from that of CK, and the root soluble protein contents of the remaining treatment groups were significantly higher than that of CK (*P*<0.05). The root soluble sugar content of T11–28 was not significantly different from that of CK, and the remaining treatment groups had significant promotion effects (*P*<0.05), among which T00–11 had the best promotion effect, with a 1.70-fold enhancement compared with CK. BC00, BV11, and T11–28 had significant promotion effects on root soluble starch content (*P*<0.05), among which BC00 had the most significant promotion effect, with a 1.35-fold enhancement compared with CK (**Figures 2A, B**).

### 3.6 Effects of endophytes on *I. indigotica* endogenous hormone

Compared with CK, the ABA contents in leaves and roots in all treatment groups was significantly lower than that of CK (*P*<0.05), IAA content in leaves in all treatment groups were significantly higher than that of CK (*P*<0.05), and GA_3_ contents in leaves in treatment groups except for T00–28 and T11–28 groups were significantly higher than that of CK (*P*<0.05), and ZR contents of leaves of BC00, BV11, and T11–28 were significantly higher than that of CK (*P*<0.05). The IAA contents in the roots of BC00, PA28, and T00–11 were significantly higher than that of CK (*P*<0.05), the GA_3_ contents in all treatment groups except for the PA28 group were significantly higher than that of CK (*P*<0.05), and the ZR contents in all treatment groups except for the T00–28 group increased significantly (*P*<0.05) (**Figures 2C–F**).

### 3.7 Effects of endophytes on *I. indigotica* active ingredient

**Figure 3:**
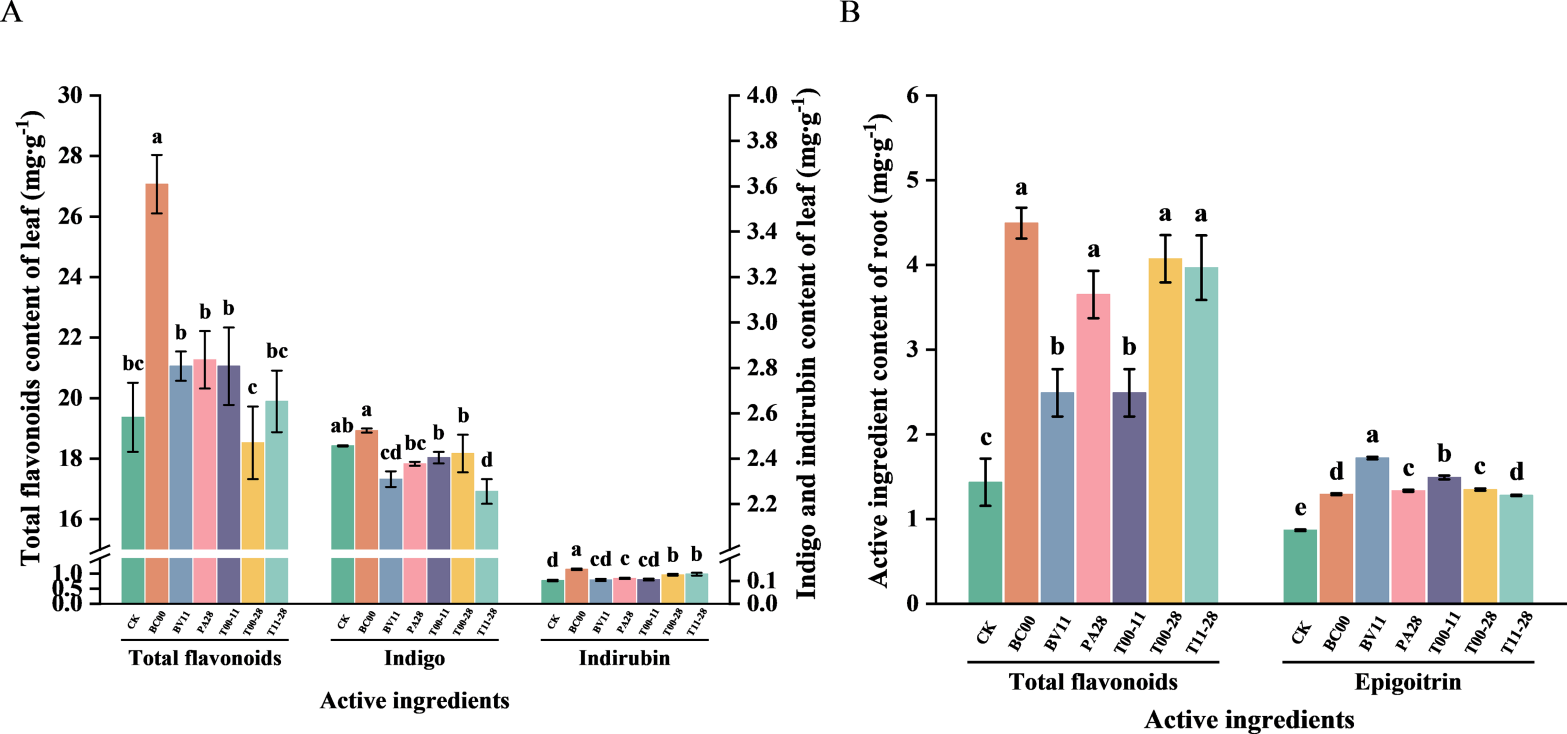
Contents of active ingredient in *I. indigotica* under different treatments (*n* = 3). **(A)** Active ingredient content in leaves, **(B)** Active ingredient content in roots. Different lowercase letters indicate statistically significant differences among groups at the p<0.05 level.

The effects of endophytic bacteria on total flavonoids, indigo, and indigo contents in the leaves were different, in which the total flavonoids and indigo contents in leaves under BC00 treatment were significantly higher than those in CK (*P* < 0.05), increasing by 39.76% and 48.57%, compared to CK, respectively. The indigo content of leaves in BC00 raised by 2.73% with CK. Compared to CK, the total flavonoids and epigoitrin in roots of all treatment groups were significantly higher than those in CK (*P* < 0.05). After treatment with BC00, the total flavonoids in the roots increased by 213.17%, and the epigoitrin in BV11 increased by 98.16%, compared to CK (**Figure 3**).

### 3.8 Effects of endophytes on *I. indigotica* rhizosphere soil chemical properties

**Figure 4:**
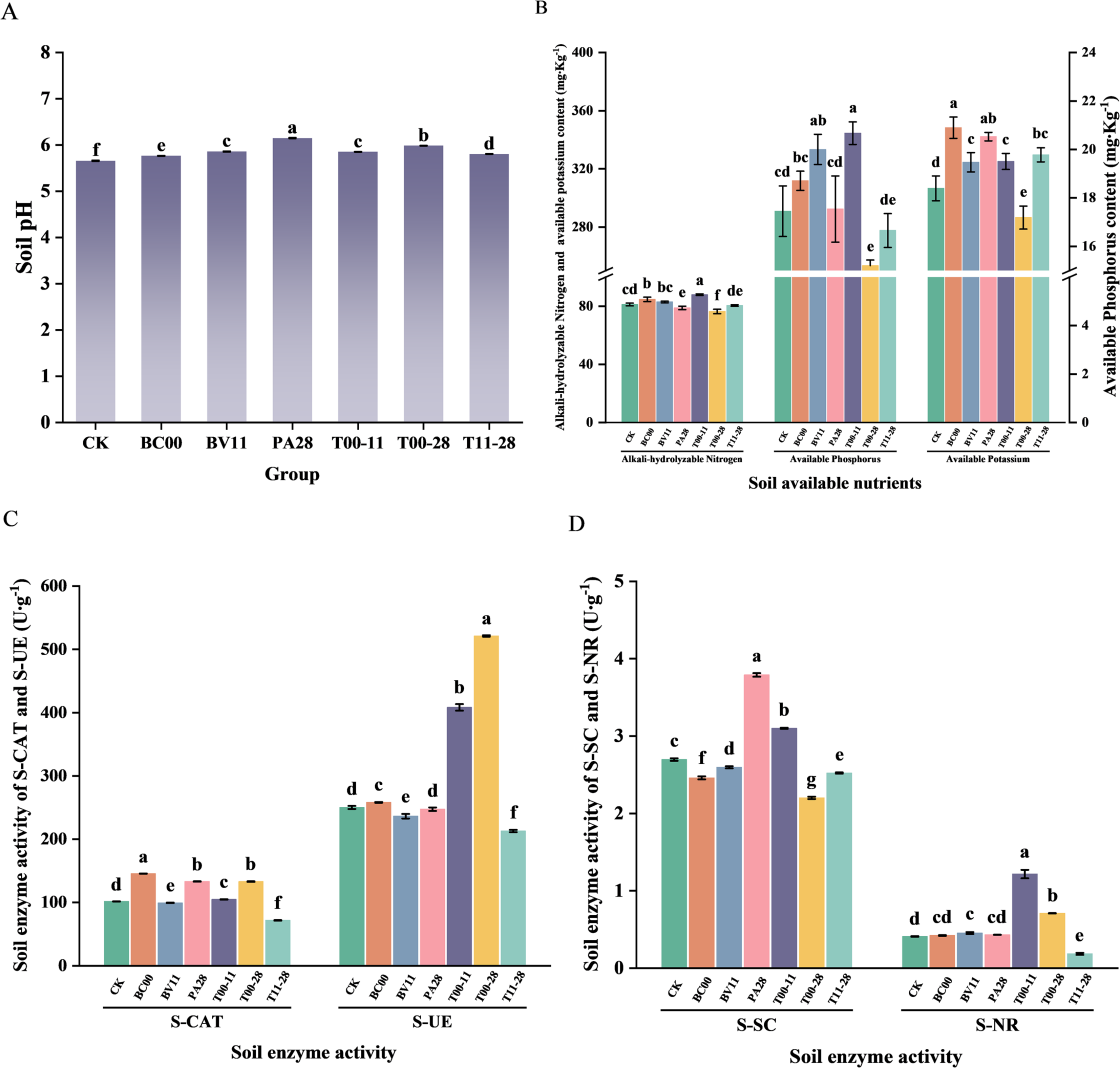
Soil chemical properties and enzyme activities under different treatments (*n* = 3). **(A)** Soil pH, **(B)** Soil nutrient content, **(C)** Soil catalase activity and soil urease activity, **(D)** Soil sucrase activity and soil nitrate reductase activity. Different lowercase letters indicate statistically significant differences among groups at the p<0.05 level.

The soil pH was 5.66 without the application of endophytic bacteria, and after endophytic bacteria treatment, the soil pH was significantly increased (*P*<0.05), and the soil acidity was reduced, in which PA28 treatment was more neutral. BC00, BV11, and T00–11 had significant promotion effects on alkali-hydrolyzable nitrogen and available phosphorus contents (*P*<0.05), and the most significant effect was observed in T00-11, which was 8.31% and 18.46% higher than that of CK, respectively. Except for the T00–28 group, the contents of available potassium in all treatment groups were significantly higher than that of the CK (*P*<0.05) except for the T00–28 group, in which BC00 had the best promotion effect, with a 13.61% increase compared with CK (**Figures 4A, B**).

### 3.9 Effects of endophytes on *I. indigotica* rhizosphere soil enzyme activities

Except for BV11 and T11-28, the rhizosphere soil catalase activities of all treatment groups were stronger than that of CK (*P*<0.05), and BC00 had the most significant promotion effect, with a 1.43-fold increase compared with CK. BC00, T00-11, and T00–28 had a significant promotion effect on soil urease activity (*P*<0.05), with 1.03-fold, 1.63-fold, and 2.08-fold increases compared with that of CK, respectively. Compared with CK, the soil sucrase activities of PA28 and T00–11 were significantly stronger than CK (*P*<0.05), and increased 1.41 times and 1.15 times, respectively, and except for the T11–28 group, all the other treatment groups had a significant promotion effect on soil nitrate reductase activity (*P*<0.05), among which T00–11 had the best promotion effect and increased 2.97 times (**Figures 4C, D**).

### 3.10 Correlation analysis between soil properties and plant traits

**Figure 5:**
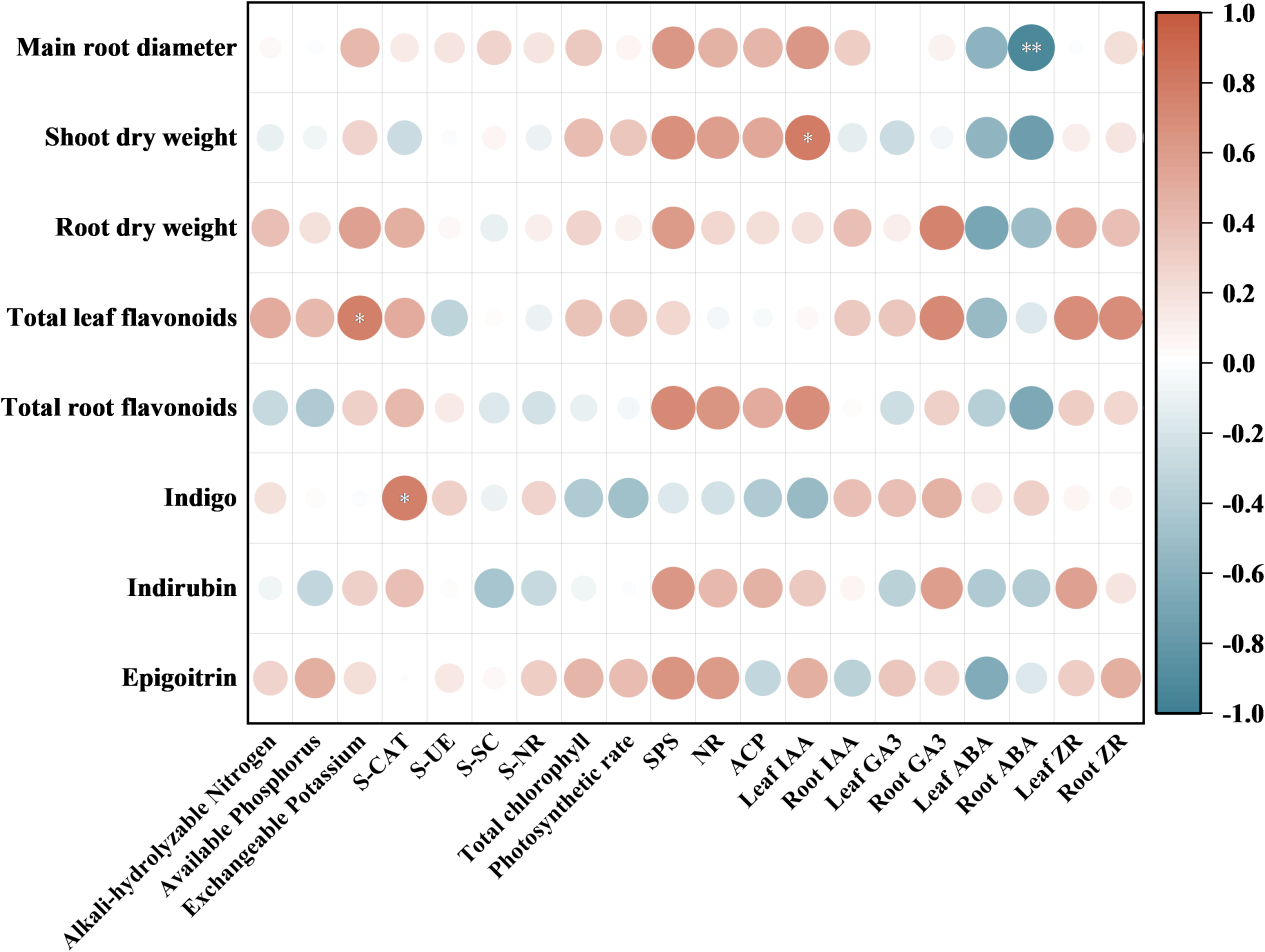
Correlation heatmap of soil properties and plant traits. * *P*<0.05, ** *P*<0.01.

Soil nutrients, soil enzyme activities, total chlorophyll content, net photosynthetic rate, key enzymes of nutrient metabolism, and endogenous hormone contents were correlated with the diameter of the main root, dry weight of shoot and root, total flavonoid content, and content of each index component (**Figure 5**). Soil available potassium content and total flavonoid content of *I. indigotica* leaves showed a significant positive correlation with each other (*P*<0.05), and soil catalase activity and indigo content of *I. indigotica* leaves also showed a significant positive correlation (*P*<0.05). The IAA content of *I. indigotica* leaves showed a significant positive correlation with the shoot dry weight (*P*<0.05), and the ABA content of *I. indigotica* roots showed a highly significant negative correlation with the diameter of *I. indigotica* roots (*P*<0.01).

### 3.11 The comprehensive analysis

To comprehensively evaluate the effects of processing with *I. indigotica* endophytic bacteria on its growth and active compound accumulation, calculate the total active biomass (**Table 4**). Compared with the CK group, the total active biomass per plant of every treatment group showed increases separately by 272.37%, 187.30%, 205.30%, 230.70%, 196.97%, and 263.32%, in which BC00 demonstrated the most significant effect in promoting the growth and active compound accumulation, with a total active biomass per plant of 87.272 mg. Because total active biomass integrates both plant yield and medicinal quality, we used it as the criterion to select the optimal treatment for further microbiome analysis. Therefore, root microbiome analysis was conducted between the BC00 treatment group and CK to preliminarily analyze the interaction mechanism among “endophytic bacteria BC00- rhizosphere microorganisms- *I. indigotica*.”

**Table 4:** Total active biomass of single plant of *I. indigotica* (mean ± SD, *n* = 3).

Treatment	Total epigoitrin/mg	Total Indigo/mg	Total indirubin/mg	Total active ingredient/mg
CK	4.755 ± 0.054f	17.933 ± 0.015e	0.749 ± 0.025e	23.437 ± 0.023g
BC00	43.369 ± 0.380a	41.415 ± 0.150d	2.488 ± 0.064b	87.272 ± 0.483a
BV11	19.080 ± 0.165e	46.154 ± 0.686c	2.101 ± 0.092d	67.335 ± 0.685f
PA28	20.699 ± 0.180d	48.573 ± 0.183b	2.281 ± 0.060c	71.553 ± 0.364d
T00-11	29.844 ± 0.454b	45.637 ± 0.482c	2.023 ± 0.080d	77.505 ± 0.410c
T00-28	20.964 ± 0.205d	46.208 ± 1.581c	2.430 ± 0.072bc	69.601 ± 1.445e
T11-28	22.427 ± 0.156c	59.302 ± 1.434a	3.422 ± 0.167a	85.151 ± 1.344b

Different lowercase letters indicate statistically significant differences among treatments at the p<0.05 level.

### 3.12 Rhizosphere soil bacterial community comparison

#### 3.12.1 Alpha diversity analysis

The curves tended to flatten as the sequence count increased, and the sequencing depth was reasonable. The Sobs index and Shannon index of the BC00 group were smaller than that of CK, indicating that its richness and diversity were smaller than that of the CK, but there was no significant difference (**Supplementary Figure 1**).

**Figure 6:**
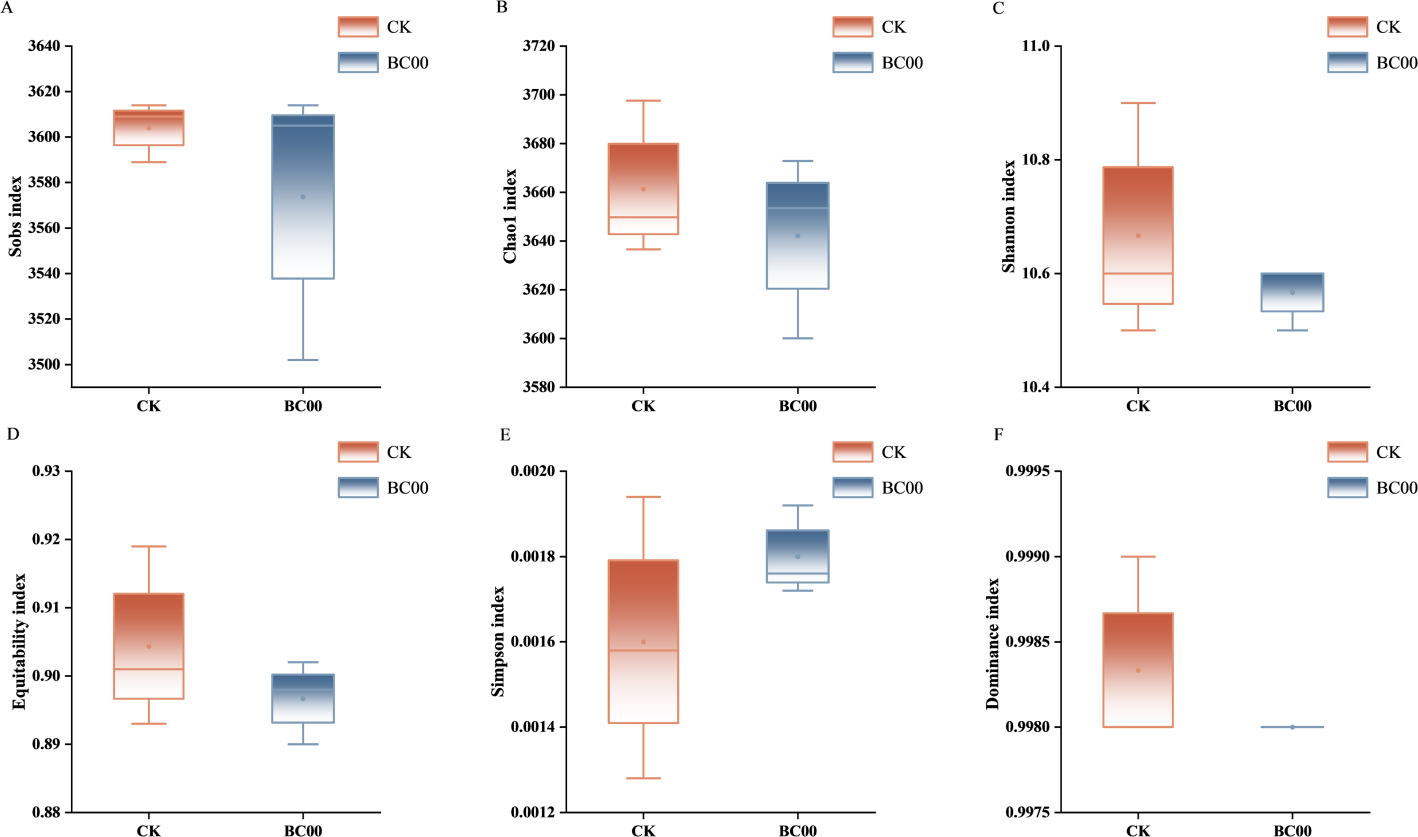
Difference analysis of Alpha diversity index of rhizosphere soil bacterial community under different treatments. **(A)** Sobs, **(B)** Chao1, **(C)** Shannon, **(D)** Equitability, **(E)** Simpson, **(F)** Dominance, Different colors represent different treatment groups.

The Sobs index and Chao1 index represent the number of the actually observed species and the estimated total number of bacterial species in the community, respectively. The Sobs index and Chao1 index of the BC00 treatment group were smaller than those of CK, which indicated that its richness was smaller. The indices reflecting the diversity of the community are the Shannon index and Simpson index. The Shannon diversity index increases with greater community diversity. Conversely, the Simpson index yields lower values when diversity is higher. The BC00 treatment group’s Shannon index was smaller than CK’s, and the Simpson index was larger than CK’s, which showed that the diversity of its bacterial community was lower than that of CK. The equitability index reflects the species evenness, the closer the value was to 1, the more uniform the distribution of species was. The dominance index reflects the degree of species dominance, the higher the value, the more obvious the dominant species. The equitability index and dominance index of BC00 treatment groups were smaller than that of CK, which indicated that the CK species distribution was more uniform and the dominant species was more obvious. Except for the dominance index, there was no significant difference between BC00 treatment and CK (**Figure 6**).

#### 3.12.2 Principal co-ordinates analysis

Principal Coordinates Analysis (PCoA) based on the Bray-Curtis distance algorithm (**Supplementary Figure 2**). Principal components PC1 and PC2 explained 31.8% and 22.6% of the sample dissimilarity, respectively, which the total numbers of PC1 and PC2 were 54.4%. The samples from BC00 and CK were significantly separated at the PC1 (31.8%) level, indicating that the rhizosphere soil bacterial communities between the BC00 treatment group and CK were different, but there was no significant difference.

#### 3.12.3 Multi-level bacterial community comparison and DESeq2 analysis

**Figure 7:**
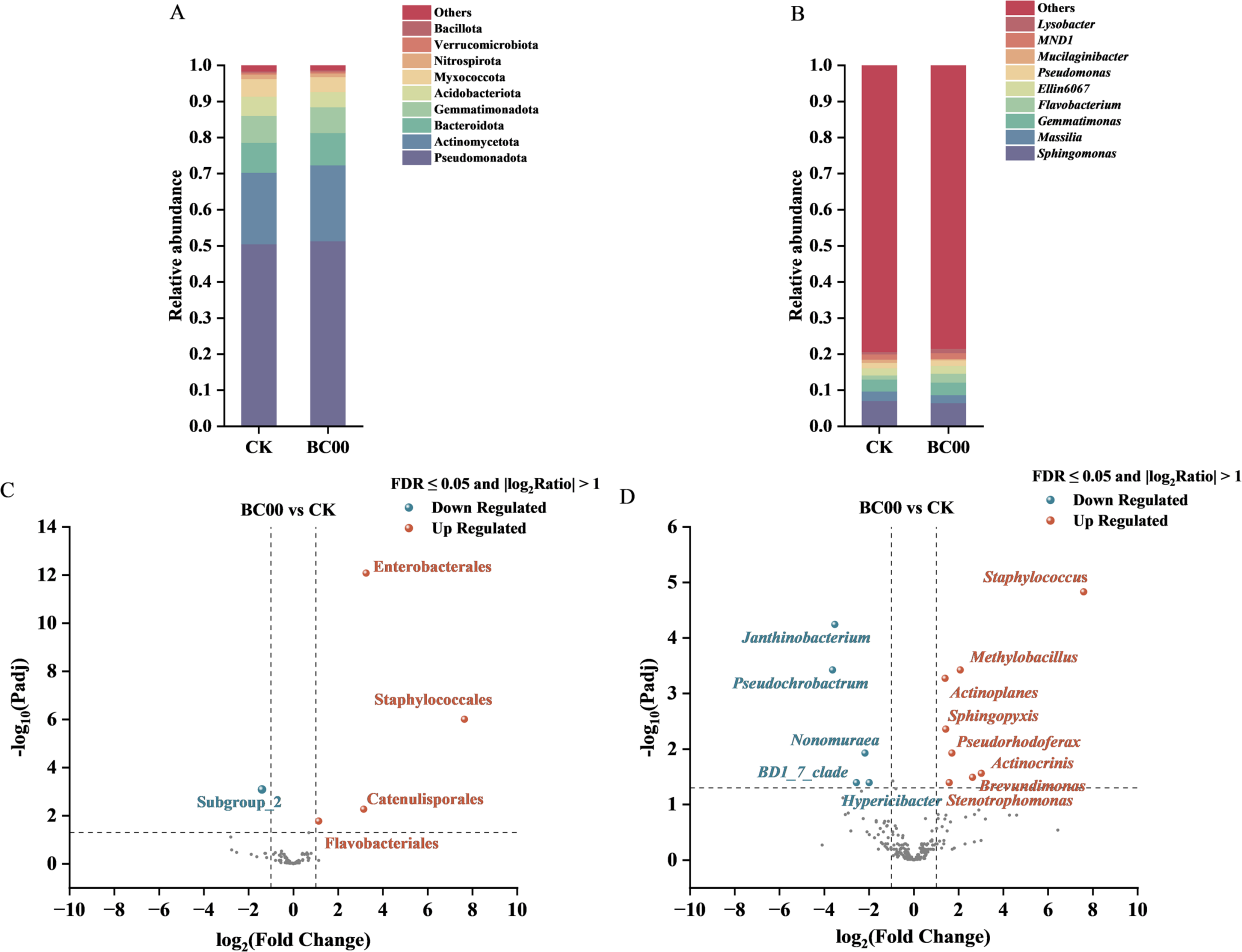
Multi-level analysis of rhizosphere bacterial communities under different treatments. **(A)** Relative abundance of dominant bacterial phyla, **(B)** Relative abundance of dominant bacterial genera, **(C)** Differentially abundant bacterial orders identified by DESeq2, **(D)** Differentially abundant bacterial genera identified by DESeq2, Different colors represent different bacteria.

Generally, the relative abundance greater than or equal to 1% was generally defined as the dominant bacterial flora (**Figures 7A, B**). Pseudomonadota, Actinomycetota, Bacteroidota, Gemmatimonadota, Acidobacteriota, Myxococcota, and Nitrospirota were co-dominant bacterial phyla in BC00 and CK. *Sphingomonas*, *Massilia*, *Gemmatimonas*, *Flavobacterium*, *Ellin6067*, *Pseudomonas*, and *Mucilaginibacter* are co-dominant bacterial genera in BC00 and CK. Compared with CK, *Lysobacte*r was the unique dominant genus for BC00.

Differential taxa between BC00 and CK were identified by DESeq2 analysis (**Figures 7C, D**). Compared to CK, at the order level, Enterobacterales, Staphylococcales, Catenulisporales, and Flavobacteriales were significantly up-regulated, and Subgroup_2 was significantly down-regulated in the BC00 group, and at the genus level, *Staphylococcus*, *Methylobacillus*, *Actinoplanes*, *Sphingopyxis*, *Pseudorhodoferax*, *Actinocrinis*, *Brevundimonas*, and *Stenotrophomonas* were significantly up-regulated, and *Janthinobacterium*, *Pseudochrobactrum*, *Nonomuraea*, *BD1_7_clade*, and *Hypericibacter* were significantly down-regulated in the BC00 group.

#### 3.12.4 Picrust 2 bacterial community functional prediction

**Figure 8:**
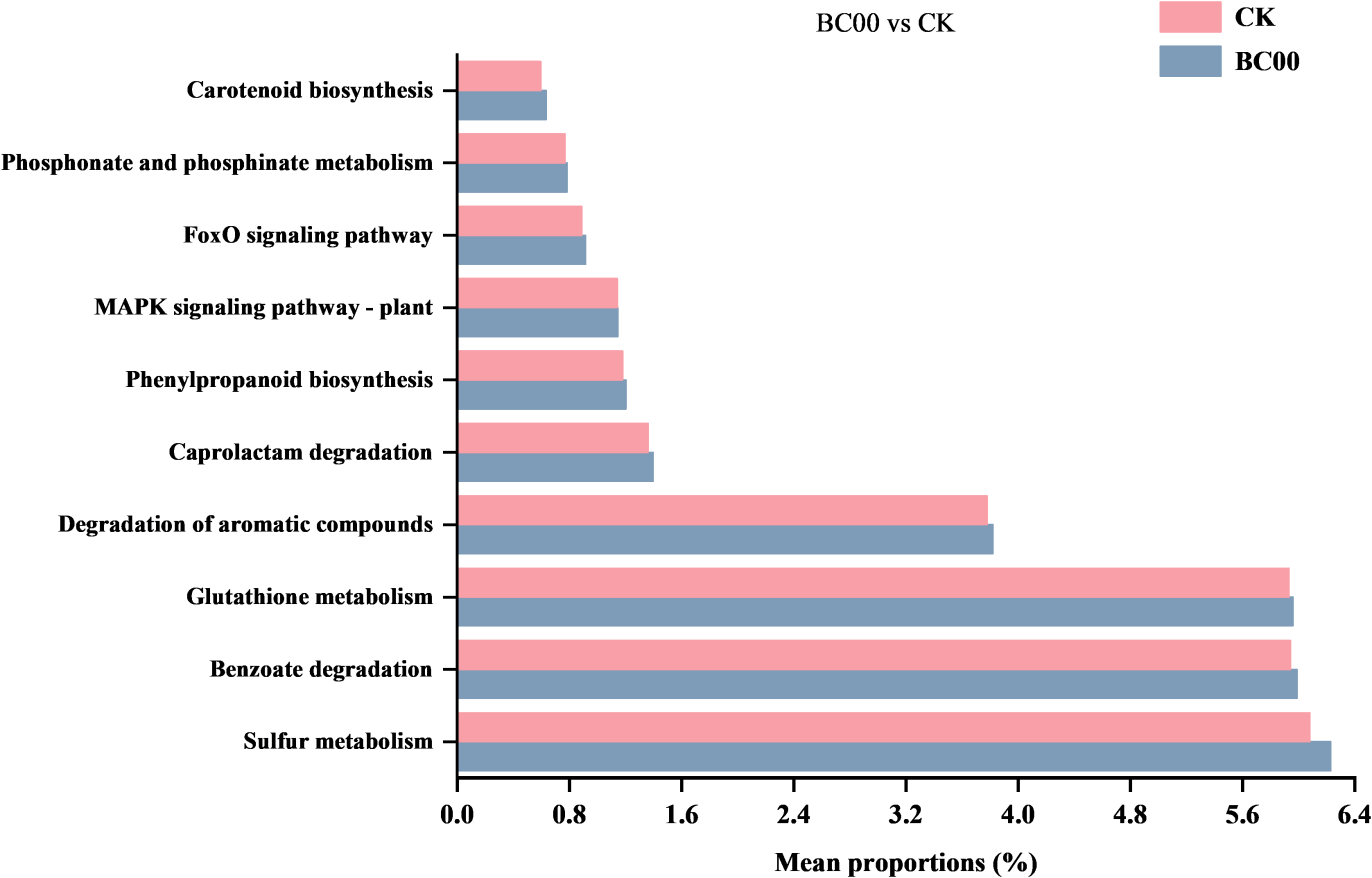
Predicted functional potential of the bacterial community. Different colors represent different treatment groups.

The functional genes of the microbial community were analyzed and predicted by Picrust 2, and these genes were mapped to specific metabolic pathways to screen out the metabolic pathways that were superior to CK in BC00 rhizosphere soil bacteria and compare the relative abundance of the BC00 and CK (**Figure 8**). The endophytic bacterium BC00 treatment promoted multiple metabolic pathways, including sulfur metabolism, benzoate degradation, glutathione metabolism, degradation of aromatic compounds, caprolactam degradation, phenylpropanoid biosynthesis, MAPK signaling pathway, FoxO signaling pathway regulation, phosphate and phosphinate metabolism, and carotenoid biosynthesis.

### 3.13 Rhizosphere soil fungi community comparison

#### 3.13.1 Alpha diversity analysis

The curves tended to flatten as the number of sequences increased, and the amount of sequencing was reasonable. The Sobs and Shannon indices of BC00 treatment groups were smaller than those of CK, indicating that their richness and diversity were smaller than those of the CK, but there was no significant difference (**Supplementary Figure 3**).

**Figure 9:**
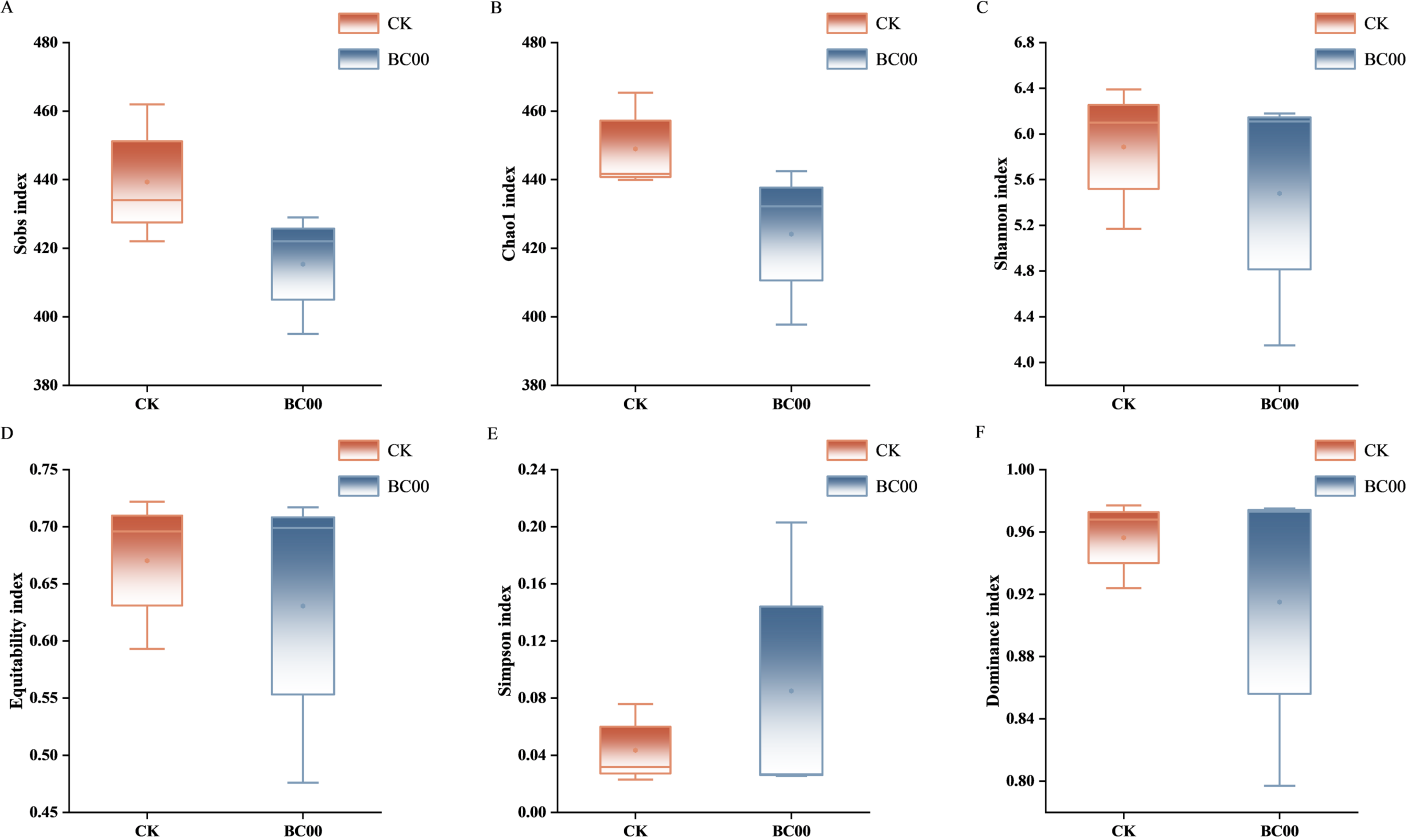
Difference analysis of Alpha diversity index of rhizosphere soil fungus community under different treatments. **(A)** Sobs, **(B)** Chao1, **(C)** Shannon, **(D)** Equitability, **(E)** Simpson, **(F)** Dominance, Different colors represent different treatment groups.

The Sobs index and Chao1 index of the BC00 treatment group were smaller than those of CK, which indicated that its richness was smaller; the BC00 treatment group’s Shannon index was smaller than CK’s, and the Simpson index was larger than CK’s, which showed that the diversity of its fungal community was lower than that of CK. The equitability index and dominance index of the BC00 treatment group were both smaller than CK, which indicated that the CK species distribution was more uniform and the dominant species were more obvious. However, there was no significant difference in each index (**Figure 9**).

#### 3.13.2 Principal co-ordinates analysis

Principal Coordinates Analysis (PCoA) based on the Bray-Curtis distance algorithm (**Supplementary Figure 4**). Principal components PC1 and PC2 explained 40.2% and 33.2% of the sample dissimilarity, respectively, which totaled 73.4%. The samples from BC00 and CK were significantly separated at the PC1 (40.2%) level, indicating that the rhizosphere soil fungal communities between the BC00 treatment group and CK were different, but there was no significant difference.

#### 3.13.3 Fungi community comparison and DESeq2 analysis at phylum and genus levels

**Figure 10:**
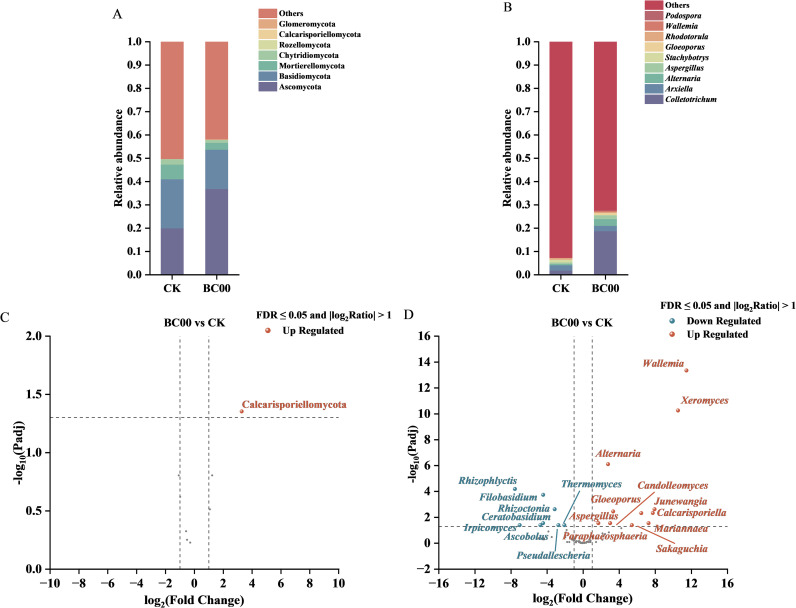
Multi-level analysis of rhizosphere fungal communities under different treatments. **(A)** Relative abundance of dominant fungal phyla, **(B)** Relative abundance of dominant fungal genera, **(C)** Differentially abundant fungal orders identified by DESeq2, **(D)** Differentially abundant fungal genera identified by DESeq2, Different colors represent different fungi.

Ascomycota, Basidiomycota, Mortierellomycota, and Chytridiomycota were the common dominant phyla in BC00 and CK; *Colletotrichum* and *Arxiella* were the BC00 and CK co-dominant genera, *Alternaria* and *Aspergillus* are BC00 exclusive dominant genera, and *Stachybotrys* is the CK exclusive dominant genus (**Figures 10A, B**).

Compared to CK, at the phylum level, Calcarisporiellomycota was significantly up-regulated in the BC00 group, and at the genus level, *Wallemia*, *Xeromyces*, *Alternaria*, *Junewangia*, *Gloeoporus*, *Calcarisporiella*, *Candolleomyces*, *Mariannaea*, *Sakaguchia*, *Paraphaeosphaeria*, and *Aspergillus* were significantly up-regulated, and *Rhizophlyctis*, *Filobasidium*, *Rhizoctonia*, *Ceratobasidium*, *Irpicomyces*, *Ascobolus*, *Pseudallescheria*, and *Thermomyces* were significantly down-regulated in the BC00 group (**Figures 10C, D**).

### 3.14 “Endophyte-rhizosphere microbe-plant” interaction analysis

**Figure 11:**
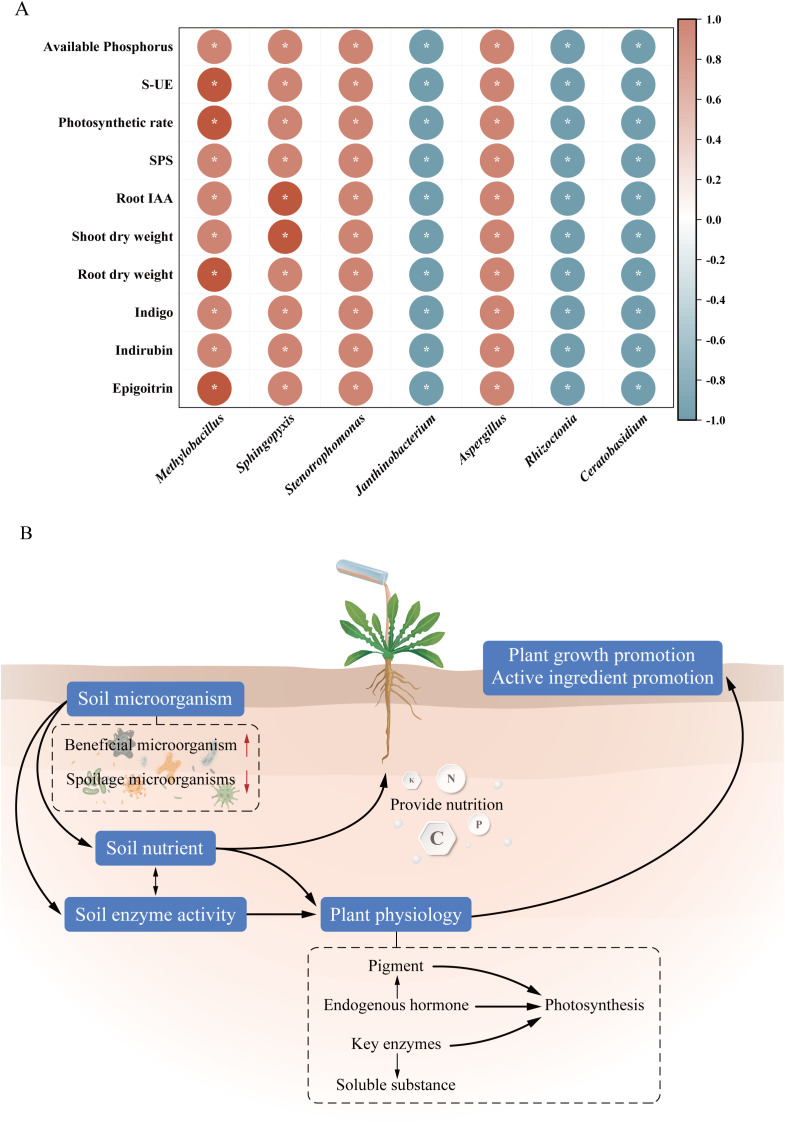
**(A)** Correlation analysis between key differential microbial genera and soil-plant indicators, *P<0.05. **(B)** Endophytic bacteria-microbial community structure-plant growth physiological interaction network diagram of *I. indigotica*.

Selected key differential microbial genera, including *Methylobacillus*, *Sphingopyxis*, *Stenotrophomonas*, *Janthinobacterium*, *Aspergillus*, *Rhizoctonia*, and *Ceratobasidium*, and soil-plant indicators, including soil available phosphorus, S-UE, photosynthetic rate, SPS, root IAA, shoot and root dry weight, and the content of each index component, were subjected to correlation analysis (**Figure 11A**). Among these, *Methylobacillus*, *Sphingopyxis*, *Stenotrophomonas*, and *Aspergillus* showed significant positive correlations with the above indicators (*P*<0.05), while *Janthinobacterium*, *Rhizoctonia*, and *Ceratobasidium* showed significant negative correlations (*P*<0.05).

The endophytic bacteria of *I. indigotica* can influence the physiological metabolism by regulating the structure of the microbial community, which in turn can promote the growth and the synthesis of active ingredients of *I. indigotica* (**Figure 11B**). After the treatment of the bacterial suspension, the soil properties and enzyme activities were influenced by the enrichment of beneficial microorganisms and the down-regulation of harmful microorganisms, which further affected the physiological metabolism and provided energy and material basis, regulated cell division and elongation, and balanced the nutrient distribution in *I. indigotica* through the promotion of photosynthesis, the enhancement of the content of endogenous hormones, and the regulation of the carbon and nitrogen metabolism, which ultimately promoted growth and the synthesis of active ingredients of *I. indigotica*.

## 4 Discussion

Endophytes and rhizosphere microorganisms have positive regulatory effects on plants, such as promoting plant growth, enhancing plant stress resistance, remediating contaminated soils, and promoting the accumulation of secondary metabolites (Feng et al., 2025). The endophytes can also induce changes in the structure of the soil microbial community (Berg et al., 2021), and the “endophyte-microbe-plant” is an interaction network that is worth exploring. Currently, studies in medicinal plants such as *Lycium barbarum*, *Astragalus membranaceus*, and *Glycyrrhiza uralensis* have demonstrated that endophytic bacteria affect the rhizosphere flora to varying degrees, thereby promoting plant growth; fewer studies have been carried out on the endophytic bacteria of *I. indigotica*, and there are no clear reports on their potential to promote growth and their ability to improve the rhizosphere environment.

### 4.1 Plant physiological responses to endophytes

Endophyte treatments affected the physiological network of the host plant *I. indigotica* through several pathways. It affected plant photosynthesis by increasing chlorophyll content, which was significantly increased in the BV11 group, whose photosynthetic capacity similarly rose. Enhanced photosynthetic capacity can directly promote plant growth. Treatment with 3R:1B red-blue composite light in *I. tinctoria* resulted in enhanced photosynthesis, with significant increases in plant height, root length, and biomass (Zhang et al., 2025). In this study, the photosynthetic rates of BC00 and T11–28 were significantly increased, and their plant height and various biomass parameters were also significantly increased. There is a dynamic equilibrium between photosynthesis and plant carbon, nitrogen, and phosphorus metabolism, and photosynthetic products directly drive the metabolism and allocation of carbon, nitrogen, and phosphorus, while the supply of carbon, nitrogen, and phosphorus feedback regulates photosynthetic efficiency (Bangari and Nataraja, 2024; Wang et al., 2019; Lu et al., 2025). The endophytic bacteria of *Achnatherum inebrians* improved the growth of the plant under high salt concentration by increasing the photosynthetic capacity and the activity of nitrogen-metabolizing enzymes (Bangari and Nataraja, 2024). In this study, it was found that endophytic bacterial treatment could significantly increase the activities of SPS and NR, the key enzymes required for carbon and nitrogen metabolism in the growth process of *I. indigotica*, and BC00, PA28, and T11–28 could also significantly increase the activity of ACP, and its photosynthetic capacity was also more obviously enhanced compared with other treatment groups. Carbon and nitrogen metabolism can promote the synthesis of soluble proteins, and the SPS and NR activities of cucumber were significantly increased after inoculation with *Bacillus subtilis* SX13, and its soluble protein accumulation was also significantly elevated (Wang et al., 2022). In this study, it was found that, except for the BC00 root soluble protein, which was not significantly different from that of CK, the contents of soluble proteins in roots and leaves of the rest of the treatment groups were significantly increased. The endophytic bacteria *Bacillus subtilis* BD24-2, *Pseudomonas aeruginosa* TD33-1, and *Pseudomonas aeruginosa* RD7–4 treatments of Moso Bamboo (*Phyllostachys edulis*) significantly increased the accumulation of soluble sugar and soluble starch contents (Zhao et al., 2023). In our study, the endophytic bacterial treatments inhibited the synthesis of soluble sugar in *I. indigotica* leaves, which soluble sugar is the substrate of starch synthesis, and maybe due to the conversion of soluble sugar into starch. Some studies have also pointed out that there is a certain negative correlation between starch content and soluble sugar content (Chen and Zhao, 2007; Xu et al., 2011). In addition, endophyte treatment may induce plant defense responses, increasing the consumption of soluble sugars for the synthesis of defense-related substances such as phenolics and flavonoids (Liu et al., 2022), or alter carbon allocation through sugar signaling pathways, prioritizing the distribution of carbon flux to secondary metabolism. This shift in carbon metabolism may provide the material basis for the endophyte-promoted accumulation of bioactive compounds in *I. indigotica*.

Endophytic bacteria can directly benefit host plants by regulating relevant plant hormones (Afzal et al., 2019). IAA and ABA can further affect plant photosynthesis by regulating stomatal opening, and ZR can delay leaf senescence and reduce chlorophyll degradation. The endophytic *Bacillus thuringiensis* strains NB49 and Bt12, isolated from *Salvia miltiorrhiza*, promoted the accumulation of zeatin while inhibiting that of ABA (Ma et al., 2025). The endophytic bacterium *Kocuria rosea* can colonize the root system of *Rehmannia glutinosa* and stimulate the secretion of IAA and zeatin (Wang et al., 2024). In the present study, we found that the ABA content of leaves and roots of *I. indigotica* was significantly lower than that of CK with the endophytic bacteria treatment. However, the contents of IAA, GA_3_, and ZR were both promoted and inhibited, which was hypothesized to be probably due to the complex interactions and equilibrium between different hormones in the plant (Munné-Bosch and Müller, 2013).

### 4.2 Plant growth and active ingredient responses to endophytes

As an emerging microbial resource, endophytic bacteria of Chinese medicinal herbs show broad application prospects in many fields such as agricultural cultivation, industrial production, medicine and health, and ecological environment protection (Golinska et al., 2015; Cui et al., 2016) and mainly promote plant growth through mechanisms such as nitrogen fixation, production of phytohormones, and production of siderophores (Aishwarya et al., 2024). Under drought stress, *Glycyrrhiza uralensis* inoculation with *Bacillus amyloliquefaciens* FZB42 significantly promoted root elongation, and the concentration of the bacterial solution, 10^7^ CFU·mL^-1^, was more effective for biomass accumulation (Yue et al., 2022). In this study, the phenotypic parameters such as plant height, main root diameter, and dry and fresh weight of *I. indigotica* showed significant improvement after endophytic bacterial treatments, in which T11–28 was more conducive to the growth of shoot parts, while BC00 was more favorable to the growth of roots. Endophytes can induce the synthesis of active ingredients in Chinese herbal medicines, such as *Salvia miltiorrhiza* inoculated with *Pseudomonas aeruginosa* Pse145 significantly increased the content of medicinal active ingredients, such as tanshinone I and salvianolic acid B (Guo et al., 2025), and dark septate endophytes (DSE) increased the content of epigoitrin in the roots of *I. indigotica* under drought stress (Li et al., 2023). In this study, the endophyte treatments increased the contents of indirubin in leaves and epigoitrin in roots of *I. indigotica*, with BC00 and BV11 being the most significant, respectively, while the indigo content in leaves was slightly lower than that of CK in all treatment groups except for the BC00 group. Indigo and indirubin are isomers with common precursors, isatan and indican (Yang et al., 2010), and the indigo content was slightly lower than that of CK after part of the endophytic bacterial treatments, which was presumed to be because the precursor substances were converted to indirubin. However, after comprehensively analyzing the contents of biomass and the index components, it was found that the total active biomass of single plants of *I. indigotica* was significantly increased after the treatment of endophytic bacterial treatments. This indicated that both the single application of three strains of endophytes and the combination of two endophyte strains are beneficial to the growth and increasing medicinal quality of *I. indigotica*.

### 4.3 Rhizosphere soil properties and enzyme activities responses to endophytes

Endophytic bacteria improve the rhizosphere environment by affecting rhizosphere soil nutrient content and enzyme activities. It has been shown that the plant-promoting bacterium *Bacillus* sp. RC01 can significantly affect soil total mineral nitrogen and nitrate nitrogen content, as well as soil available phosphorus content (Canbolat et al., 2006). In the present study, the *Bacillus* sp. BC00, BV11, and the two-bacteria composite T00–11 groups significantly promoted alkali-hydrolyzable nitrogen and available phosphorus content, and the available potassium content of all the treatment groups, except for the T00–28 group, was significantly higher than that of CK. *Pseudomonas putida* was able to increase the activities of soil urease, phosphatase, and invertase (Nosheen et al., 2018). Our study found that different endophytic bacterial treatments had different degrees of influence on soil enzyme activities, and BC00 and T00–11 had the most significant effect on the promotion of soil catalase and soil nitrate reductase activities, respectively, with the former decreasing the hydrogen peroxide toxicity to plant roots by maintaining the redox balance of the soil and plant roots, and the latter reducing nitrate toxicity to plant roots through the maintenance of the soil and plant root redox balance. BC00, T00-11, and T00–28 significantly promoted soil urease activity, catalyzing the hydrolysis of urea to ammonia and CO_2_, which is the key enzyme of the nitrogen cycle. PA28 and T00–11 soil sucrase activity was significantly stronger than that of CK, decomposing sucrose to glucose and fructose and directly providing carbon sources for plants.

### 4.4 Rhizosphere soil microbial diversity responses to endophytes

Endophyte treatments may promote plant growth by regulating the structure of rhizosphere soil microbial communities, enriching beneficial microorganisms, and reducing pathogenic microorganisms. It has been shown that The application of *Bacillus subtilis* and biochar not only increased the fungal diversity in the rhizosphere soil of *Panax notoginseng*, but also suppressed pathogenic fungi such as *Fusarium* (Zhou et al., 2024), inoculation of peanut (*Arachis hypogaea* L.) with two strains of *Bacillus beleriensis* RI3 and SC6 did not significantly change the microbial diversity but altered the relative abundance of dominant phylum and genus (Bigatton et al., 2024), and under salt stress, inoculation of tomato plants with *Bacillus* sp. H19–1 and H20–5 did not significantly differ in their effects on microbial diversity and abundance, and both alpha diversity indices were slightly reduced after 21 d of H20–5 treatment (Yoo et al., 2021). In this study, the difference in rhizosphere soil microbial diversity after *Bacillus* sp. BC00 treatment was not significant, and the abundance and diversity were slightly reduced compared with the CK, and there were differences in the relative abundance of bacterial and fungal co-dominant flora at the phylum and genus levels. DESeq2 analysis of differentially abundant species revealed that some beneficial microorganisms were significantly up-regulated after BC00 treatment, and some phytopathogenic microorganisms were significantly down-regulated. Further integrated correlation analysis indicated that the upregulated genera *Methylobacillus*, *Sphingopyxis*, *Stenotrophomonas*, and *Aspergillus* showed significant positive correlations with soil available phosphorus, S-UE, photosynthetic rate, SPS, root IAA, shoot and root dry weight, and the content of each index component. The genus *Methylobacillus* comprises methylotrophic bacteria with nitrogen-fixing capabilities that contribute to carbon and nitrogen cycling in soil (Kumar et al., 2019). *Sphingopyxis* possess genetic potential for IAA synthesis and exhibit biocontrol activity against harmful microorganisms (Sharma et al., 2021). *Stenotrophomonas* demonstrates multiple functions, including phosphorus solubilization, nitrogen fixation, and IAA production (Kumar et al., 2023), while *Aspergillus* species significantly enhance plant biomass by improving photosynthetic and nutrient utilization efficiencies (Sidhoum et al., 2024). Endophytic bacterium BC00 promotes growth and secondary metabolite accumulation in *I. indigotica* by enriching multifunctional beneficial microbial communities, activating rhizosphere soil nutrients, and regulating phytohormone synthesis and photosynthetic carbon metabolism. Concurrently, this beneficial shift was accompanied by a marked suppression of pathogenic fungi, including *Rhizoctonia* and *Ceratobasidium*, which are known agents of root rot and seedling blight (Zeng et al., 2023; English et al., 1986). The endophytic bacterium BC00 may be associated with reduced disease risks in *I. indigotica*, potentially linked to the suppressing harmful pathogens in rhizosphere soil, which could contribute to enhanced yield and medicinal quality. Soil microbial community has certain stability, and different soil microorganisms may perform similar functions, and endophyte treatment may only affect a few specific functional groups, but the overall diversity indexes do not change significantly. Even if the relative abundance of some groups changes, other taxa may quickly fill the ecological function vacancies, resulting in insignificant differences in diversity indexes.

### 4.5 Comparison and application selection between single-strain and combination treatments

In this study, the effects of single-strain and combination treatments on the growth and active ingredient accumulation in *I. indigotica* were compared. The results showed that single-strain treatments exhibited relatively specific functions, BC00 was more beneficial to root growth and total biomass accumulation, BV11 significantly increased photosynthetic rate and chlorophyll content, and PA28 showed the greatest promoting effects on leaf number and lateral root number. Combination treatments exhibited synergistic effects on certain parameters, T11–28 favored shoot growth and significantly increased ACP activity, T00–11 showed notable effects in promoting chlorophyll a, carotenoids, root soluble sugar, and soil alkali-hydrolyzable nitrogen and available phosphorus, while T00–28 significantly increased SPS and NR activities. In terms of total active biomass, the single-strain treatment BC00 performed best, with total active biomass significantly higher than that of the other treatments. This result suggests that in the development of microbial inoculants for *I. indigotica*, a single strain with a prominent function may have greater application potential than combination treatments, owing to its clear function and its ability to maximize total active biomass. The advantages of combination treatments lie in their complementary functions and balanced overall performance; however, the interactions among different strains are complex, and the stability of their effects may be influenced by strain ratios and environmental conditions. In addition, in this study, none of the combination treatments surpassed the optimal single-strain treatment in terms of total active biomass. Therefore, in practical applications, when the core objective is to promote root development or increase total active biomass, single strains with prominent functions such as BC00 can be prioritized. When a balanced improvement of both shoot and root growth is desired, or when multiple metabolic pathways need to be simultaneously activated, combination treatments may offer greater reference value.

Comprehensive analysis revealed that the differences in rhizosphere soil microbial diversity after endophyte treatment were not obvious and similar in species composition. However, it may activate the metabolic activity of functional flora, leading to the up-regulation of some of the flora related to nutrient cycling, which in turn altered the soil nutrient content and enzyme activity and also improved the rhizosphere environment through the up-regulation of some of the flora capable of degrading organic pollutants. The increase in soil fertility provided nutrients for the plant and further promoted the growth of *I. indigotica* and the synthesis of active ingredients by influencing photosynthesis, increasing the activity of key enzymes of nutrient metabolism, and synthesizing endogenous hormones.

## 5 Conclusions

In summary, three endophytic bacterial strains (*Bacillus* sp. BC00, *Bacillus* sp. BV11, and *Pseudomonas* sp. PA28) of *I. indigotica*, single endophytic bacterial strain and combination of every two bacterial strains treatments, could regulate the growth and physiological processes of *I. indigotica* by improving the rhizosphere soil environment, which in turn affects the synthesis of its leaf and root active ingredients. The *Bacillus* sp. BC00 was the most effective in promoting the growth of *I. indigotica*, and the dominant endophyte BC00 was associated with altered structure of the rhizosphere microbial community, which may relate to the observed growth and active ingredient synthesis. Although the present study demonstrates the beneficial effects of endophytic bacteria on growth and active ingredient accumulation in *I. indigotica*, the underlying molecular mechanisms remain to be elucidated, which constitutes a limitation of this work. Subsequent work could employ fluorescence labeling tracing to clarify the colonization process of dominant endophytic bacteria, utilize metatranscriptomics for functional verification of the rhizosphere microbial community, and reveal its regulatory mechanisms on primary and secondary metabolism in *I. indigotica* through integrated transcriptomic and metabolomic analyses.

## Data Availability

The datasets presented in this study can be found in online repositories. The names of the repository/repositories and accession number(s) can be found below: https://www.ncbi.nlm.nih.gov/, PRJNA1430213.
