# Supplementary material for: The effect of endophytic bacteria on the growth, medicinal quality, and rhizosphere soil environment of Isatis indigotica Fort
Source: Front Plant Sci. 2026 Apr 22;17:1821717. doi: 10.3389/fpls.2026.1821717 (PMC13144005; doi:10.3389/fpls.2026.1821717)
Supplement: Supplementary file 1 [file DataSheet1.docx]

Supplementary Material

**1 Supplementary Figures**


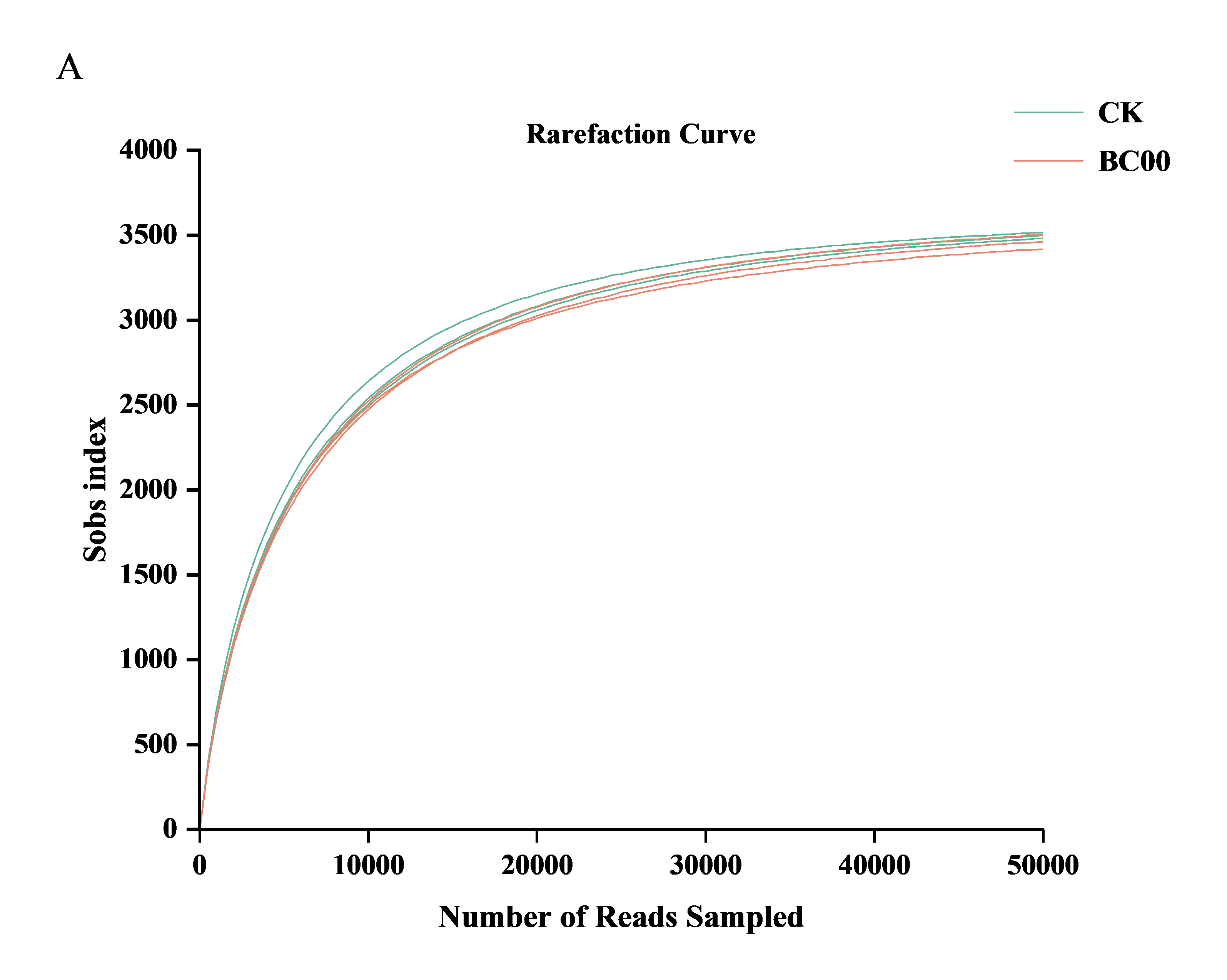

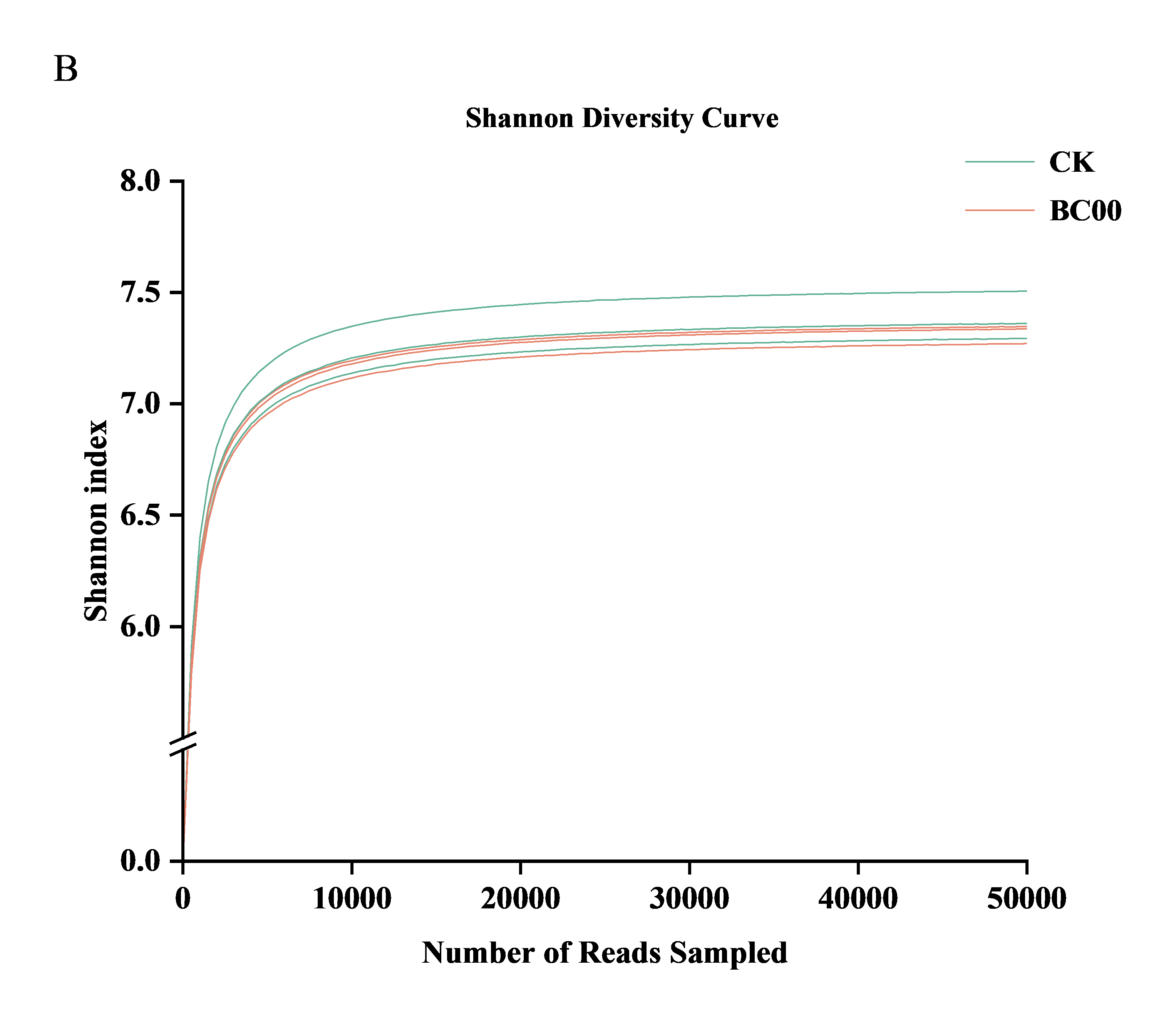


Supplementary Figure 1. Analysis of sampling depth and alpha diversity. (A) Rarefaction curve, (B) Shannon diversity curve, Different colors represent different treatment groups.


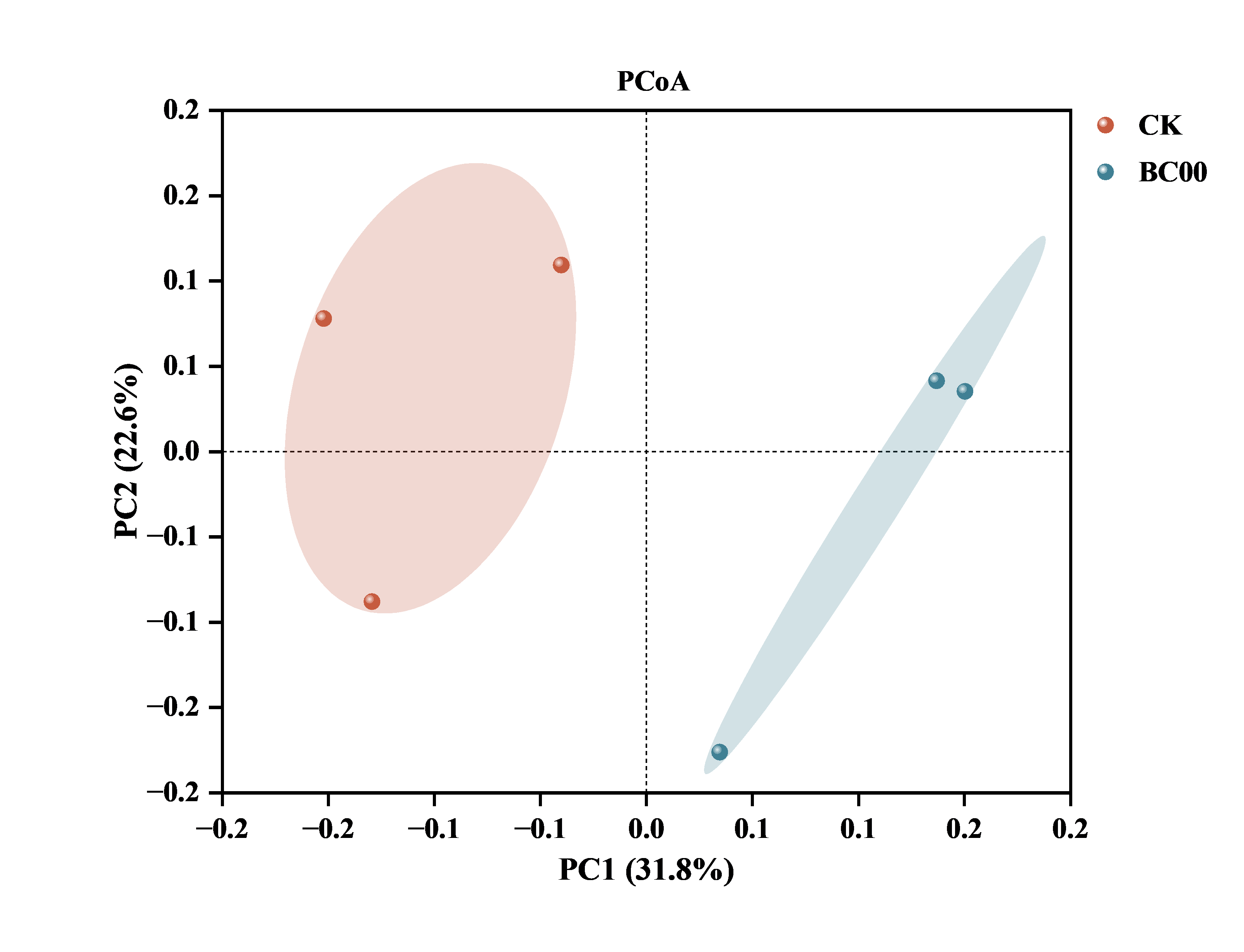


Supplementary Figure 2. Principal coordinate analysis of rhizosphere soil bacterial community under different treatments. Different colors represent different treatment groups.


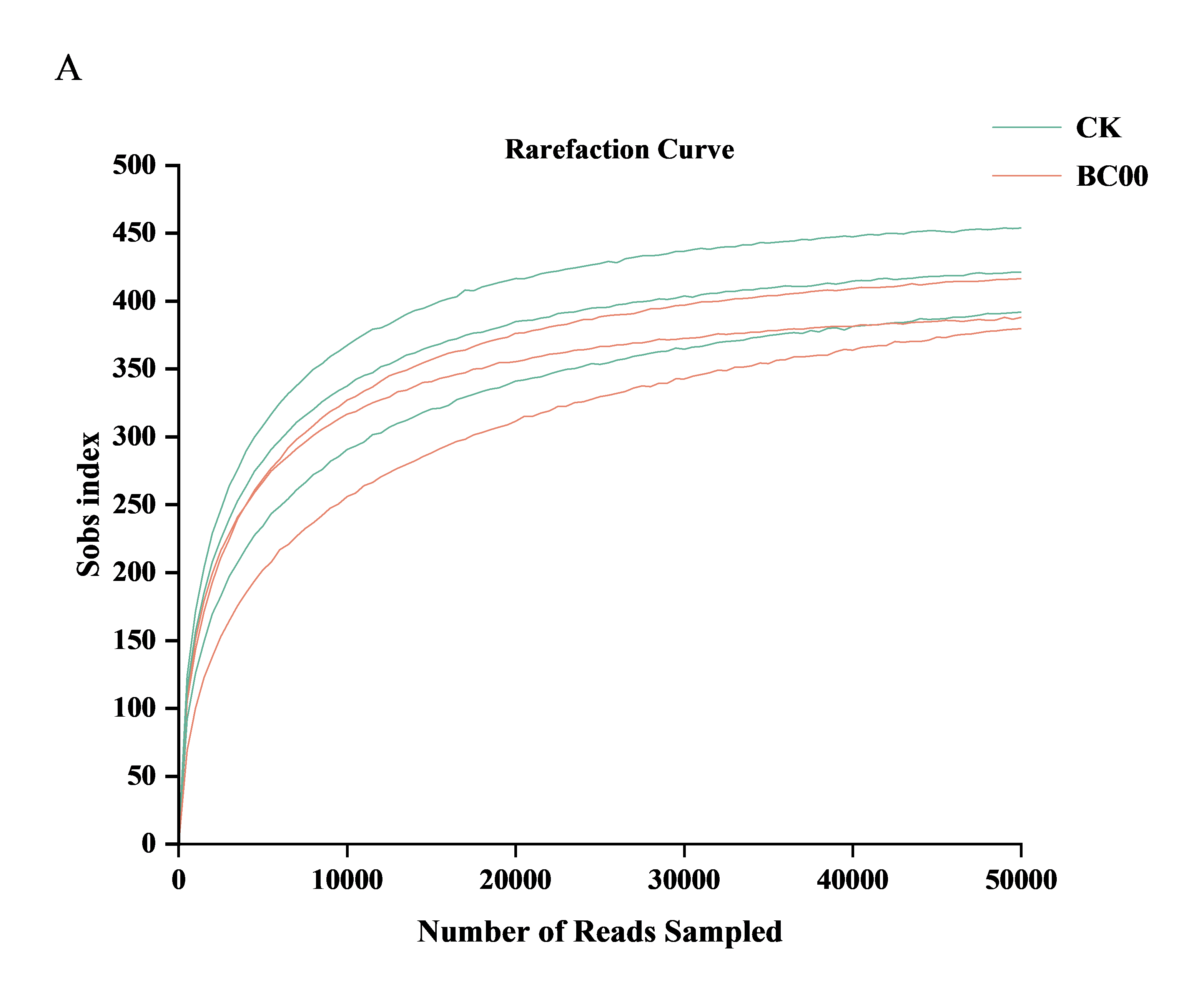

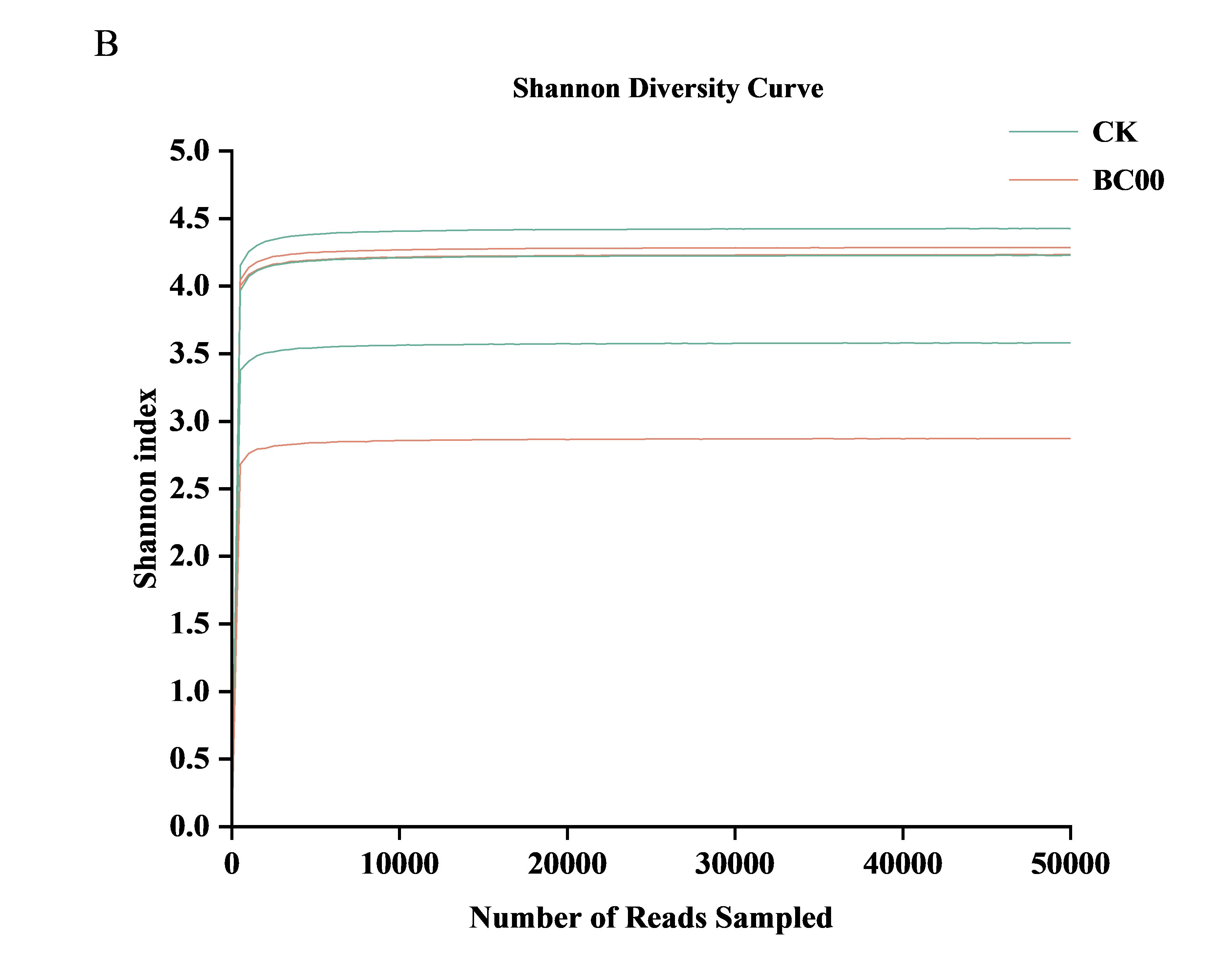


Supplementary Figure 3. Analysis of sampling depth and alpha diversity. (A) Rarefaction curve, (B) Shannon diversity curve, Different colors represent different treatment groups.


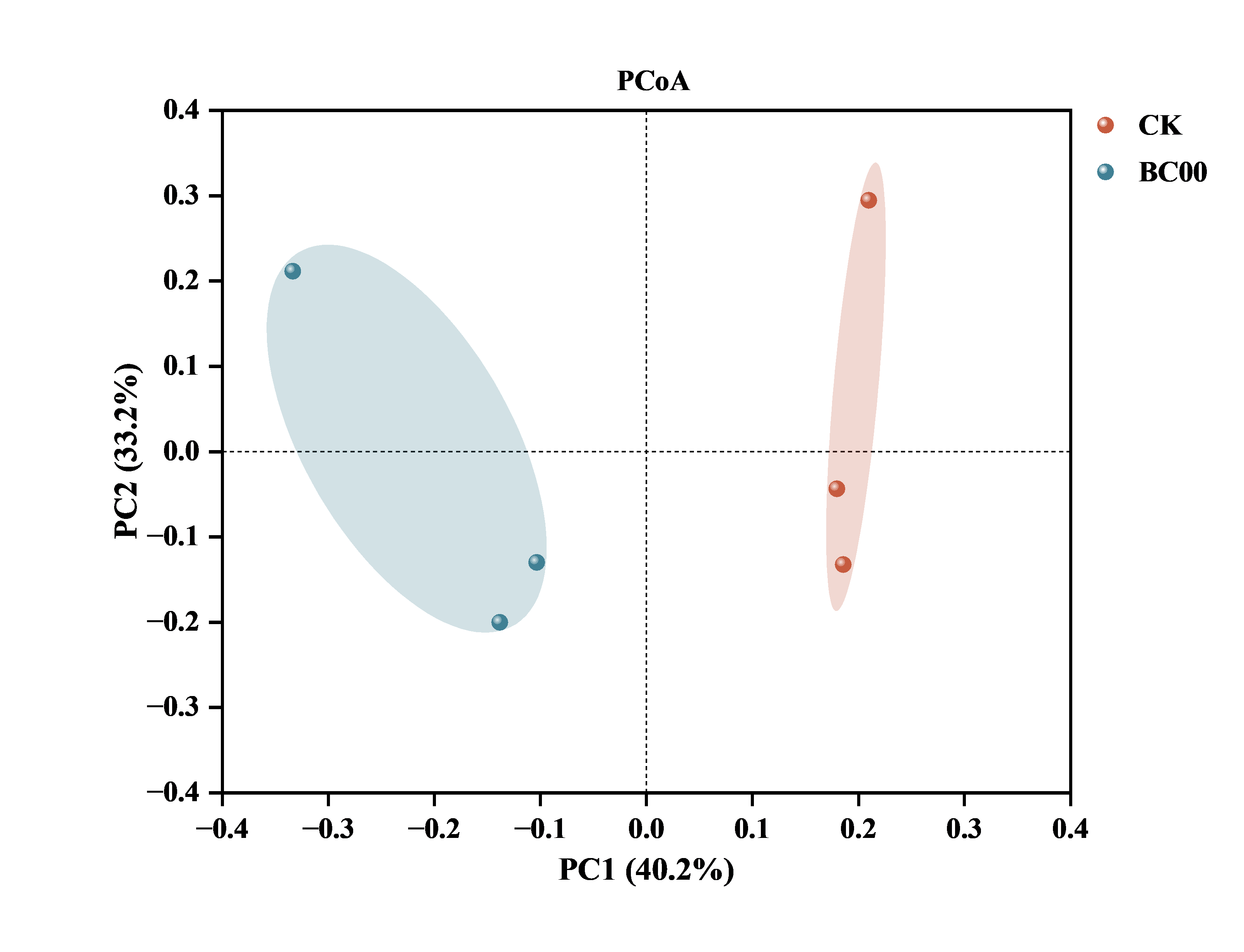


Supplementary Figure 4. Principal coordinate analysis of rhizosphere soil fungus community under different treatments. Different colors represent different treatment groups.
